# Using a Hybrid Mapping Population to Identify Genomic Regions of *Pyrenophora teres* Associated With Virulence

**DOI:** 10.3389/fpls.2022.925107

**Published:** 2022-06-23

**Authors:** Buddhika A. Dahanayaka, Lislé Snyman, Niloofar Vaghefi, Anke Martin

**Affiliations:** ^1^Centre for Crop Health, University of Southern Queensland, Toowoomba, QLD, Australia; ^2^Department of Agriculture and Fisheries Queensland, Hermitage Research Facility, Warwick, QLD, Australia; ^3^School of Agriculture and Food, University of Melbourne, Parkville, VIC, Australia

**Keywords:** hybrids, quantitative trait loci, barley, candidate genes, leaf symptoms, net-form net blotch, spot-form net blotch

## Abstract

Net blotches caused by *Pyrenophora teres* are important foliar fungal diseases of barley and result in significant yield losses of up to 40%. The two types of net blotch, net-form net blotch and spot-form net blotch, are caused by *P. teres* f. *teres* (*Ptt*) and *P. teres* f. *maculata* (*Ptm*), respectively. This study is the first to use a cross between *Ptt* and *Ptm* to identify quantitative trait loci (QTL) associated with virulence and leaf symptoms. A genetic map consisting of 1,965 Diversity Arrays Technology (DArT) markers was constructed using 351 progenies of the *Ptt/Ptm* cross. Eight barley cultivars showing differential reactions to the parental isolates were used to phenotype the hybrid progeny isolates. Five QTL associated with virulence and four QTL associated with leaf symptoms were identified across five linkage groups. Phenotypic variation explained by these QTL ranged from 6 to 16%. Further phenotyping of selected progeny isolates on 12 more barley cultivars revealed that three progeny isolates are moderately to highly virulent across these cultivars. The results of this study suggest that accumulation of QTL in hybrid isolates can result in enhanced virulence.

## Introduction

*Pyrenophora teres* [syn: *Drechslera teres*] is a haploid ascomycetous pathogen that causes net blotches in barley (*Hordeum vulgare* L.). Net blotches have been reported in all barley-growing areas of the world including regions in Europe (Jonsson et al., 2000; Rau et al., 2003; Serenius et al., 2005; Bakonyi and Justesen, 2007; Ficsor et al., 2014), Middle East (Bouajila et al., 2011), Far-East (Sato and Takeda, 1997), North America (Peever and Milgroom, 1994; Jonsson et al., 2000; Akhavan et al., 2016), South America (Moya et al., 2020), South Africa (Campbell et al., 2002; Lehmensiek et al., 2010) and Oceania (Serenius et al., 2007; Bogacki et al., 2010; Lehmensiek et al., 2010; Mclean et al., 2010; Ellwood et al., 2019). Barley net blotches are economically important foliar fungal diseases worldwide with average yield losses ranging between 10 and 40% with complete destruction of plants possible in susceptible barley cultivars (Martin, 1985; Khan, 1987; Steffenson et al., 1991; Jayasena et al., 2007; Jebbouj and El Yousfi, 2009, 2010; Moya et al., 2020). In Australia alone, the potential annual economic losses due to *P. teres* have been estimated to be over AUD $300 million (Murray and Brennan, 2010).

Net blotches occur as two types based on the symptoms: net-form net blotch (NFNB) caused by *Pyrenophora teres* f. *teres* (*Ptt*) and spot-form net blotch (SFNB) caused by *P. teres* f. *maculata* (*Ptm*; Smedegård-Petersen, 1971). Symptoms caused by both *Ptt* and *Ptm* initially appear as chlorotic spots. In NFNB, chlorotic regions later extend into longitudinal and transverse net-like necrotic streaks. In SFNB, initial chlorosis develops into circular spot-like necrotic lesions. The two forms have identical morphology and can only be differentiated using molecular markers (Williams et al., 2001; Keiper et al., 2008; Poudel et al., 2017). *Pyrenophora teres* is a heterothallic fungus. Therefore, two opposite mating types are required for sexual reproduction (Mcdonald, 1963). Sexual reproduction in *P. teres* is controlled by a single mating type locus (*MAT1*), which exists as two alternative forms or idiomorphs, i.e. *MAT1*-1 and *MAT1*-2 (Mcdonald, 1963).

Molecular studies have shown that the two forms of *P. teres* are phylogenetically independent and divergent groups (Mclean et al., 2009; Liu et al., 2011; Ellwood and Wallwork, 2018; Syme et al., 2018; Marin-Felix et al., 2019; Clare et al., 2020); hence, sexual reproduction between the two forms is suggested to be rare (Serenius et al., 2005; Lehmensiek et al., 2010; Mclean et al., 2014; Akhavan et al., 2015). However, identification of *Ptt*/*Ptm* hybrids collected from barley fields (Campbell et al., 2002; Leišova et al., 2005; Mclean et al., 2014; Dahanayaka et al., 2021b; Turo et al., 2021) and successful establishment of laboratory-based hybrids (Smedegård-Petersen, 1971; Crous et al., 1995; Louw et al., 1995; Campbell et al., 1999; Jalli, 2011) suggest that the two forms can overcome sexual reproduction barriers under certain environmental conditions (Dahanayaka et al., 2021a). Hybridisation between the two forms may result in hybrids harbouring both *Ptt* and *Ptm* virulence genes, which could result in devasting yield losses in the absence of barley cultivars resistant to both *P. teres* forms. Therefore, comprehensive knowledge of possible pathotypes that could arise from a hybrid population and identification of genomic regions associated with virulence would help accelerate the development of new hybrid resistant barley cultivars.

It has been suggested that the barley-*P. teres* pathosystem fits the gene-for-gene model, where qualitative traits or dominant genes are involved in the infection process (Flor, 1956; Mode and Schaller, 1958; Weiland et al., 1999; Friesen et al., 2006, 2008; Afanasenko et al., 2007). However, with the identification of host selective toxins, also known as necrotic effectors (NEs), it was proposed that in addition to the gene-for-gene interaction, an inverse process of gene-for-gene interaction may occur in the *P. teres*-barley pathosystem through NEs-mediated programmed cell death, similar to the one found in the wheat pathogen *P. tritici repentis* (Friesen et al., 2008; Ciuffetti et al., 2010; Faris et al., 2010).

Fungal effectors are proteins that act as either avirulence/virulence factors or both (Kamoun, 2007). Pathogens have evolved to manipulate their effectors as a response to the host defence mechanism (Selin et al., 2016; Białas et al., 2018). To verify the long-term endurance of the pathogen, constant development of novel effectors may be needed to allow recognition of new host targets (Möller and Stukenbrock, 2017). In order to undergo rapid evolution and alteration of effectors according to the host response, genomic regions associated with effectors reside in low complexity regions, which often harbour transposable elements (TEs) and repeat-rich regions of the pathogen genome (Raffaele and Kamoun, 2012; Dong et al., 2015). As a result, these genomic regions show increased point mutagenesis (Rouxel et al., 2011), extensive chromosomal rearrangements and structural polymorphisms (De Jonge et al., 2013; Möller and Stukenbrock, 2017).

Recent *P. teres* secretome analyses, including *in planta* analyses, highlighted the significant role of effectors/NEs in the infection process and virulence mechanisms (Ismail and Able, 2016, 2017). Several genomic regions associated with virulence/avirulence of *P. teres* have been identified and mapped using bi-parental and genome-wide association mapping populations (Weiland et al., 1999; Beattie et al., 2007; Lai et al., 2007; Shjerve et al., 2014; Kinzer, 2015; Carlsen et al., 2017; Koladia et al., 2017; Clare et al., 2020; Martin et al., 2020). As *P. teres* is a haploid fungus, it is difficult to determine the dominance of the genes responsible for virulence/avirulence. However, some of the QTL identified in these studies reported to encode effectors/NEs in *P. teres* (Martin et al., 2020). Identification of QTL/genes associated with effectors would expand the knowledge on genomic regions that drive rapid evolution and adaptation of *P. teres*.

Both *Ptt* and *Ptm* show high pathogenic variations, challenging breeding for disease resistance (Liu et al., 2011). Pathogenic variation in *P. teres* was first recorded in 1949 with the detection of differences in pathogenicity towards different barley cultivars (Pon, 1949). Since then, a number of studies have reported complex and highly pathogenic variations among *P. teres* populations worldwide (Khan, 1982; Tekauz, 1990; Steffenson and Webster, 1992; Jonsson et al., 1997; Douiyssi et al., 1998; Wu et al., 2003; Jebbouj and El Yousfi, 2010; Mclean et al., 2010; Wallwork et al., 2016; Fowler et al., 2017). Identification of large numbers of pathotypes using a differential set of barley cultivars indicates that a number of host specific effectors are involved in the *P. teres*-barley pathosystem (Carlsen et al., 2017). This suggests that a genomic region responsible for the virulence of *P. teres* on a specific barley cultivar may not be responsible for the virulence on another barley cultivar. Hence, identification of more genomic regions associated with virulence on a large number of barley cultivars is warranted in order to understand the *P. teres*-barley pathosystem.

Previously reported bi-parental mapping studies have been conducted using mapping populations developed by crossing *Ptt/Ptt* (Weiland et al., 1999; Beattie et al., 2007; Lai et al., 2007; Shjerve et al., 2014; Koladia et al., 2017; Martin et al., 2020) or *Ptm/Ptm* isolates (Carlsen et al., 2017). Using a mapping population developed by crossing *Ptt* and *Ptm* would enable the development of a high-density genetic map due to higher frequency of polymorphism between *Ptt*/*Ptm* isolates than between *Ptt/Ptt* and *Ptm/Ptm* isolates. High-density genetic maps better facilitate the identification of candidate genes (Collard et al., 2005). Furthermore, using hybrid progeny for QTL mapping may allow the identification of QTL from both *Ptt* and *Ptm* genomes and genes responsible for the different leaf symptoms caused by *Ptt* and *Ptm*, which have not been reported previously.

To gain a comprehensive understanding of the *P. teres*-barley pathosystem, the current study was conducted using a bi-parental mapping population developed from a *Ptt/Ptm* cross. The aims of the current study were to: 1. identify genomic regions associated with virulence and leaf symptoms in *P. teres*; 2. identify candidate genes encoding predicted effector-like proteins using protein information repositories; and 3. identify different virulence levels of the hybrid population across eight barley cultivars. Gaining knowledge of the genomic regions associated with virulence and leaf symptoms of net blotch will contribute towards a better understanding of the *P. teres*-barley pathosystem.

## Materials and Methods

### Biological Materials

A hybrid population (*Pop37*) consisting of 406 isolates was developed by crossing NB63 (*Ptt*: *MAT1-2*) and HRS07033 (*Ptm*: *MAT1-1*). Crosses were made as indicated in Poudel et al. (2017). Eight barley cultivars (Clho 5791, Dampier, Flagship, Fleet, Gairdner, Grimmett, Kombar and Prior) with different levels of resistance and susceptibility to NFNB and SFNB were used in phenotyping assays for the QTL analyses. A further 12 barley cultivars (Clho 11458, Beecher, Compass, Fathom, Harbin, Keel, Navigator, RGT Planet, Rosalind, Schooner, Spartacus CL and Vlamingh) known to be resistant to either the *Ptt* or *Ptm* parent isolates were used to phenotype 10 progeny isolates which were highly virulent on the original eight barley cultivars used.

### DNA Extraction and DArTseq™

Hybrid progeny cultures stored at −80°C were grown on half-strength potato dextrose agar (PDA) medium (20 g/litre PDA; Biolab Merck, Darmstadt, Germany) at 22°C for 10 days. The DNA of the parental isolates and ascospores was extracted using the Wizard^®^ Genomic DNA Purification kit following the protocol of the supplier (Promega Corporation, Wisconsin, USA). The integrity of DNA was assessed (Dahanayaka et al., 2021b) and sent to Diversity Arrays Technology Pty. Ltd. (Canberra, ACT, Australia) for DArTseq^™^.

### PCR Amplification Using Mating Type Primer Pairs

To identify the mating type of progeny isolates, PCR screening with two mating type primer pairs amplifying *Ptt*: *MAT1-2* and *Ptm*: *MAT1-1* alleles was conducted (Lu et al., 2010). The amplified PCR products were electrophoresed on 1% agarose gel stained with GelRed^®^ and the mating type of each isolate was determined according to the amplicon size (*Ptt: MAT1-*2: 1,421 bp and *Ptm*: *MAT1-1*: 194 bp). A Chi square test was conducted to examine the segregation ratio of the marker.

### Phenotypic Evaluation and Disease Assessment

Phenotypic assessments were conducted following a completely randomised design in a controlled environment room at the University of Southern Queensland, Australia, with three replicates, as described in Martin et al. (2020). The eight barley cultivars were grown in pots with 5 cm diameter and 14 cm height. Each pot contained four plants each of four barley cultivars. These were grown at 20 ± 1°C for 14 days.

The conidial suspensions for plant inoculation were prepared as follows. Agar plugs from each isolate growing on PDA (2 mm diameter each) were grown on peanut oatmeal agar (Speakman and Pommer, 1986) plates at 15 ± 1°C under black and white fluorescent lights for 10 days to induce conidia production (Fowler et al., 2017). Conidia were recovered and diluted to 10,000 conidia/mL using a Haemocytometer (Martin et al., 2020). Three millilitres of the suspension were used for each pot. Conidial suspensions were stored at −80°C (up to 3 months) until inoculation.

Fourteen days after planting, plants in each pot were sprayed with the 3 ml of conidial suspension. Two hybrid isolates and parental isolate NB63 were used as control isolates for each cycle of inoculation to monitor differences across cycles. Inoculated pots were incubated in the dark for 48 h at 95% humidity with a temperature of 20 ± 1°C. After 48 h, plants were transferred to the controlled environment room for 9 days with diurnal light at 75% humidity with a temperature of 20 ± 1°C. Nine days after inoculation, disease severity reaction on the second leaf was scored (Tekauz, 1985) and the disease symptoms, i.e. net-like or spot-like symptoms, recorded for each isolate. For disease symptoms, net-like and spot-like symptoms were denoted as 1 and 2, respectively, and treated as nominal data during QTL mapping.

The 10 progeny isolates showing the highest virulence reaction scores on the initial eight tested barley cultivars were assessed on another 12 resistant barley cultivars following the method described above.

### Genetic Map Construction

SilicoDArT and SNP marker data resulting from DArTseq™ were qualitatively filtered using Microsoft Excel (Dahanayaka et al., 2021b). Markers with more than 10% missing data and markers non-polymorphic for the parental isolates were removed. Clonal isolates of the progeny were detected using the *clonecorrect* function in *poppr* package version 2.8.3 (Kamvar et al., 2014) in RStudio version 3.0.2 (R Core Team, 2013). Both DArTseq™ markers were grouped into linkage groups using the make linkage groups function in MapManager QTXb20 version 2.0 (Manly et al., 2001) with a *p* = 0.05 search linkage criterion. Markers were ordered using RECORD (Van Os et al., 2005). The final genetic map of the population was obtained by manual map curation (Lehmensiek et al., 2009). To confirm the order of the markers within linkage groups, marker positions of the resulting genetic map were compared with marker positions of the *Ptt* and *Ptm* reference genomes: W1-1 (BioSample SAMEA4560035 available under PRJEB18107 BioProject) and SG1 (BioSample SAMEA4560037 available under PRJEB18107 BioProject), respectively. DArTseq™ marker sequences (^˷^62 bp) were aligned with the two reference genomes using the *bowtie2* (Langmead and Salzberg, 2012) function in Galaxy.

### QTL Analysis

Disease symptom scores of the progeny isolates of the NB63 and HRS07033 cross (*Pop37*) were combined with genotypic data to detect QTL associated with *P. teres* virulence using the composite interval mapping method in Windows QTL Cartographer version 2.5 (Wang et al., 2012). Experiment-wise LOD threshold values at the 0.05 significance level were estimated based on 1,000 permutation tests for each trait (Churchill and Doerge, 1994; Doerge and Churchill, 1996). Additive effects of QTL and the phenotypic variances explained by each QTL (*R*^2^) were calculated by Windows QTL Cartographer version 2.5. The resulting QTL figures were drawn using MapChart version 2.32 (Voorrips, 2002). QTL mapping was repeated with Qgene version 4.4 (Joehanes and Nelson, 2008) to confirm the significant QTL.

The nomenclature of the identified QTL was formatted as follows: the abbreviation of the institute where the QTL were detected (University of Southern Queensland, USQ) followed by the trait that the QTL is associated with and ending with the chromosome number. Where more than one QTL was identified on the same chromosome and for the same trait, a decimal value was added to the chromosome number according to the order in which they were found along the chromosome.

### Identification of Candidate Genes

A 20 kb flanking region on either side of the marker at the peak of the QTL was used to identify candidate genes within the QTL region (Martin et al., 2020). These regions were aligned with the two respective reference annotated genome assemblies (W1-1 and SG1) in the NCBI data repository. Identified candidate genes were further analysed for predicted effector genes by EffectorP version 1 and 2 (Sperschneider et al., 2016, 2018). Candidate genes were also compared with the published gene expression profiles of net blotch in barley during the infection process for effector identification (Ismail and Able, 2016, 2017).

## Results

### Filtering of Genetic Data and Clonal Isolates

A total of 6,441 SNPs and 14,549 SilicoDArT were obtained by DArTseq™. After filtering markers for 10% missing values, segregation distortion (3:1) and non-polymorphism, 632 SNPs and 1,333 SlicoDArT markers were retained for the identification of clonal isolates and the construction of the genetic map. Out of 406 isolates, 351 hybrid isolates were unique isolates. In the sexual reproduction of filamentous ascomycetous fungi, karyogamy occurs followed by meiosis. Meiosis gives rise to four haploid unique nuclei and later these four nuclei undergo mitosis to produce eight cells/ascospores. As a result of the mitosis, each ascus contains four pairs of ascospores and the ascospores of each pair are identical (Finchman, 1971). Hence, these identical isolates were removed, and 351 unique isolates were used for the phenotypic evaluation and genetic map construction.

### PCR Amplification

The PCR amplification of the mating type gene of 351 progeny isolates revealed that 166 isolates had the *Ptt MAT1-2* idiomorph (mating type 2) and the remaining 185 carried the *Ptm MAT1-1* idiomorph (mating type 1). The segregation of the population was within the 1:1 ratio (chi square 0.74; *p* = 0.390).

### Phenotypic Evaluation and Disease Assessment

**Figure 1:**
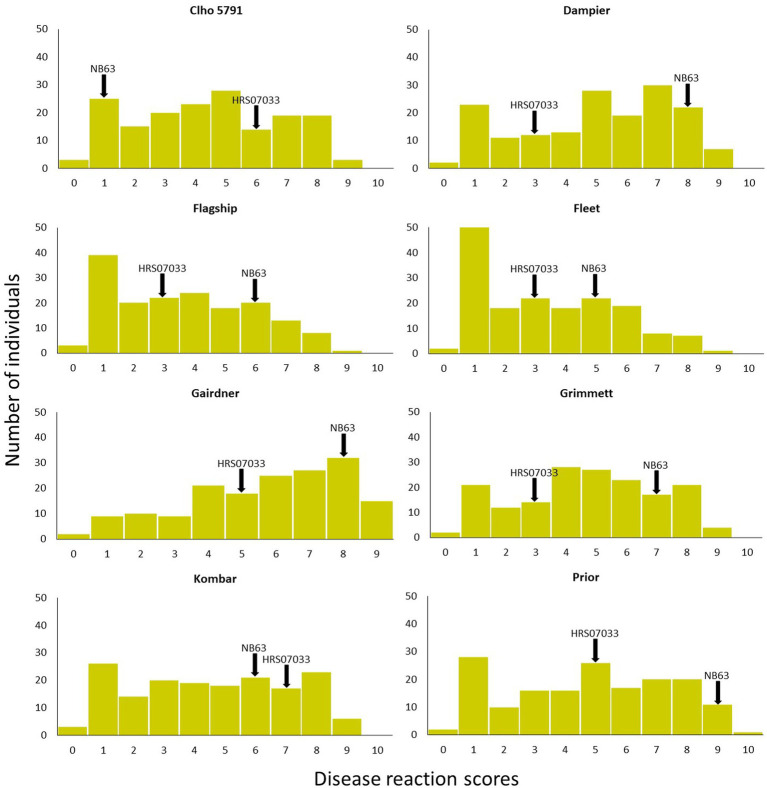
Disease reaction scores of progeny isolates of *Pop37* on eight barley cultivars/lines used in QTL analysis. Disease reaction scores of parental isolates are indicated with arrows.

**Figure 2:**
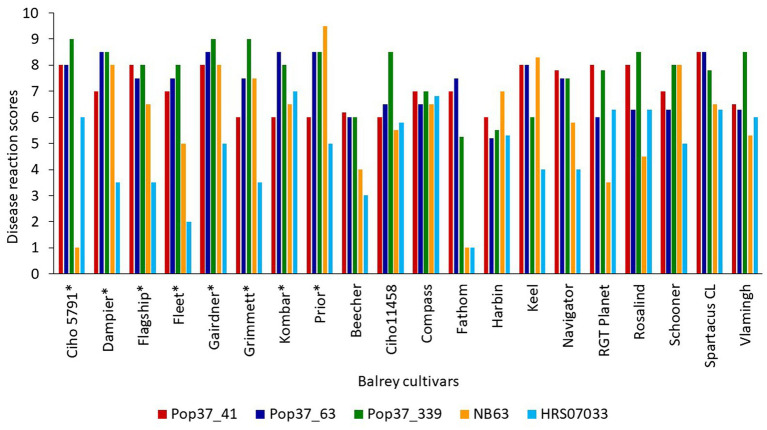
Disease reaction scores of highly virulent progeny isolates (*Pop37*_41, *Pop37*_63 and *Pop37*_339) and parental isolates (NB63 and HRS07033) of *Pop*37 population on all 20 barley cultivars/lines. ^*^Cultivars used for QTL mapping.

Out of 351 progeny isolates, 172 hybrid isolates (49%) produced conidia. Only these isolates were used for the phenotypic evaluation and in the QTL analysis. Disease reaction scores of the 172 hybrid isolates across eight barley cultivars ranged from avirulent to virulent with transgressive segregation observed for most cultivars (Table 1 and Figure 1). Of the isolates showing symptoms (*n* = 148), 13 resulted in net-like leaf symptoms and 135 in spot-like leaf symptoms. The remaining 24 isolates were avirulent (<5); thus, no symptoms were detectable on the leaves of any of the cultivars tested. Ten of the progeny isolates (*Pop37*_41, *Pop37*_48, *Pop37*_52, *Pop37*_63, *Pop37*_74, *Pop37*_237, *Pop37*_245, *Pop37*_249, *Pop37*_339 and *Pop37*_362) having scores ≥6 on all of the eight barley cultivars tested (Supplementary Table S1) were further evaluated on another 12 barley cultivars known to be resistant to either the *Ptt* or *Ptm* parental isolate. Three (*Pop37*_41, *Pop37*_63 and *Pop37*_339) of the 10 isolates had scores ≥5 on all 20 cultivars tested (Figure 2).

**Table 1:** Disease reaction scores for the eight barley cultivars/lines used for QTL analysis and virulence percentage of the hybrid population for each barley cultivar/line.

Cultivar^a^	Average^b^	SE^c^	Avirulent^d^	Virulent^e^	Virulent %^f^
Clho 5791	4.26	0.184	135	37	21.51
Dampier	4.80	0.192	124	48	27.91
Flagship	3.48	0.175	157	15	8.72
Fleet	3.17	0.173	159	13	7.56
Gairdner	3.48	0.175	157	15	8.72
Grimmett	4.56	0.181	133	39	22.67
Kombar	4.35	0.201	132	40	23.26
Prior	4.60	0.216	127	45	26.16

a Barley cultivar.

b *Average disease reaction score of progeny isolates for the respective barley cultivar.*

c *Standard error.*

d *Number of avirulent to moderately virulent isolates (< 7) out of 172.*

e *Number of highly virulent isolates (> 7) out of 172.*

f *Percentage of virulent isolates.*

### Genetic Map and QTL Analysis

Out of the original SNP and SilicoDArT markers, 1,965 high-quality markers were retained for the construction of the genetic map of NB63/HRS07033. The genetic map of *Pop37* consisted of 12 linkage groups spanning from 79.7 to 254.3 cM (Supplementary Table S2). The total length of the genetic map was 1816.3 cM with 1,432 non-redundant markers (Table 2). The average distance between flanking markers ranged from 1.152 to 1.627 per linkage group with an average distance between flanking markers of 1.268 for the entire genetic map. The physical distance to genetic map distance ratio for *Pop37* with respect to W1-1 and SG1 genomes was 28.5 kb/cM and 22.7 kb/cM, respectively. The marker order of the genetic map of *Pop37* was mostly in agreement with the marker positions of W1-1 and SG1 (Supplementary Table S2).

**Table 2:** Genetic map information of *Pop37*.

Linkage group/Chromosome	Number of markers	Non-redundant markers	Size cM	Average distance between flanking markers
1	108	86	107.9	1.255
2	203	154	200.1	1.299
3	183	120	169.7	1.414
4	154	114	136.4	1.196
5	279	212	254.3	1.200
6	67	49	79.7	1.627
7	115	86	121.2	1.409
8	276	193	230.1	1.192
9	107	79	95.6	1.210
10	96	75	97.3	1.297
11	122	81	113.2	1.398
12	255	183	210.8	1.152
Total	1965	1,432	1816.3	1.268

**Figure 3:**
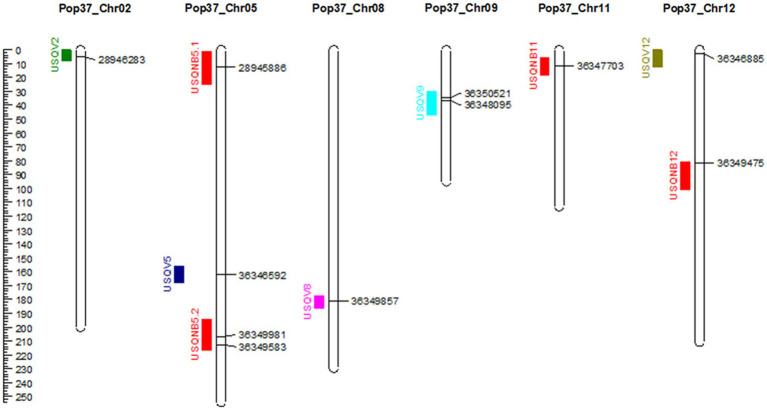
Genetic map of *Pop37* (*Ptt*-NB63 × *Ptm*-HRS07033) showing identified QTL on the left of the chromosome and markers at the peak of the QTL on the right. Distance in cM is indicated on the left.

Results of the QTL analysis using 172 hybrid progeny isolates are presented in Table 3 and Figure 3. Significant threshold LOD values based on 1,000 permutations for each barley cultivar and leaf symptom trait ranged from LOD 2.7 to 3.1. Five QTL associated with virulence on different barley cultivar were identified. The QTL *USQV12* was associated with Dampier, Grimmett, Kombar and Prior phenotypes with LOD values of 5.5, 3.3, 7.0 and 5.7, respectively. The phenotypic variation explained by this QTL was 13, 8, 16 and 14%, for Dampier, Grimmett, Kombar and Prior, respectively. The QTL *USQV9*, identified on chromosome 9, was associated with the disease reaction on Clho 5791 and Flagship with LOD scores of 3.2 and 3.6, and explained 7 and 8% of the phenotypic variance, respectively. Both QTL *USQV9* and *USQV12* were contributed by the *Ptm* parent HRS07033. The QTL *USQV2* was responsible for the variation in the disease reaction score of Flagship and Kombar with LOD 3.0 and LOD 3.8, respectively, and explained 7 to 8% of the variation in the disease reaction score of Flagship and Kombar, respectively. The QTL, *USQV8* was responsible for the variation in disease reaction score on Gairdner and had a LOD score of 3.4 explaining 10% of the phenotypic variance. The QTL, *USQV5* was responsible for the variation in disease reaction score of Fleet with LOD score of 3 and explained 6% of the phenotypic variance. The QTL *USQV2*, *USQV5* and *USQV8* were contributed by the *Ptt* parent NB63.

**Table 3:** List of virulence and leaf symptom QTL identified using *pop37*.

QTL^a^	Trait^b^	Chr^c^	Genetic map^d^				W1-1^e^			SG1^f^			LOD^g^	R^2^^h^	Parent^i^
			Start^j^ cM	End^k^ cM	Peak^l^ cM	Marker name^m^	Start (bp)	End (bp)	Peak marker (bp)	Start (bp)	End (bp)	Peak marker (bp)			
Virulence QTL															
*USQV2*	Flagship	2	1	8	2	NA	280936	346503	337069	125561	192984	NA	3.0	7	*Ptt*
	Kombar	2	1	7	6	28946283	280936	346503	337069	125561	192984	182038	3.8	8	*Ptt*
*USQV5*	Fleet	5	156	168	162.9	36346592	3626679	3745439	3719823	3043251	3161843	3136235	3.0	6	*Ptt*
*USQV8*	Gairdner	8	178	187	180	36349857	5999571	6189805	6007171	4711299	4919725	4768710	3.4	10	*Ptt*
*USQV9*	Clho 5,791	9	31	42	37	36348095	987772	1196189	1171258	734586	901217	876223	3.2	7	*Ptm*
	Flagship	9	31	48	35	36350521	987772	1273721	1055386	734586	978963	876223	3.6	8	*Ptm*
*USQV12*	Dampier	12	1	12	1 and 11	36348695	361269	508637	508637	63893	213609	128854	5.5	13	*Ptm*
	Grimmett	12	1	13	1	36346885	361269	508637	361269	63893	213609	128854	3.3	8	*Ptm*
	Kombar	12	1	12	2	36346885	361269	508637	361269	63893	213609	128854	7.0	16	*Ptm*
	Prior	12	1	11	1 and 11	36348695	361269	508637	508637	63893	213609	128854	5.7	14	*Ptm*
Leaf symptom QTL															
*USQNB5.1*	Form	5	2	25	13	28945886	256820	700266	440934	224725	470158	313831	3.2	7	*Ptm*
*USQNB5.2*	Form	5	195	217	207 and 213	36349981 & 36349583	4709550	5579160	5100294 5,151,650	3768230	4530001	4362659 4413961	3.9	9	*Ptm*
*USQNB11*	Form	11	6	18	12	36347703	200745	593148	241491	250568	394230	318802	3.4	7	*Ptm*
*USQNB12*	Form	12	78	91	78	36349475	2328147	2630739	2328147	1916192	2219650	1916192	3.0	7	*Ptm*

a *Name of the QTL.*

b *Barley cultivar used in phenotyping.*

c *Chromosome number according to W1-1 and SG1 reference genomes.*

d *Pop37 genetic map information.*

e *W1-1 reference genome.*

f *SG1 reference genome.*

g *Logarithm of the odds.*

h *Phenotypic variation described by the respective QTL.*

i *Parental isolate contributing the QTL.*

j *Starting position of the QTL.*

k *Ending position of the QTL.*

l *Peak position of the QTL.*

m *Peak position marker name of the QTL.*

**Figure 4:**
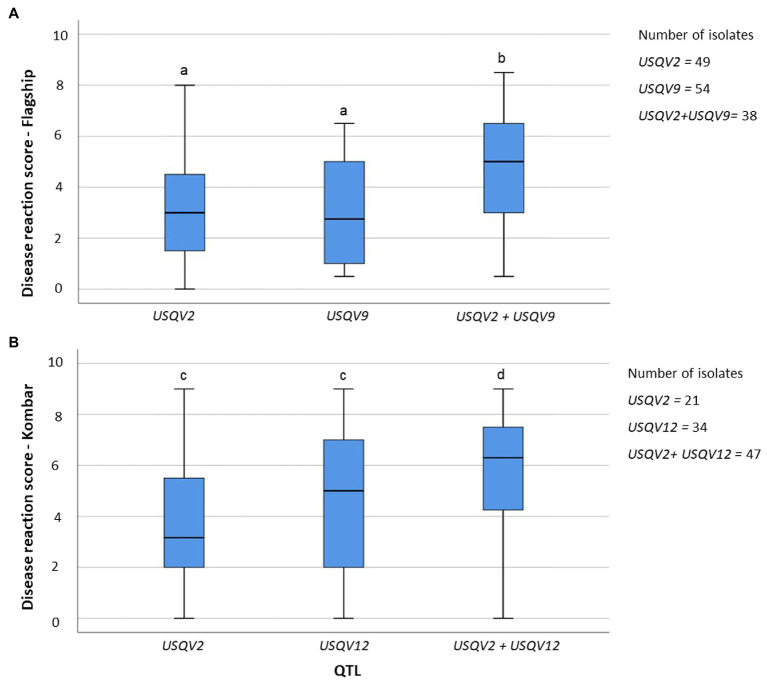
Pyramiding of QTL associated with virulence of *P. teres* for Flagship **(A)** and Kombar **(B)**. Boxes with similar letters are not significantly different (*p* = 0.05).

Out of eight barley cultivars only Flagship and Kombar were associated with more than two QTL; hence, QTL accumulation effects of progeny isolates on Flagship (*USQV2* and *USQV9*) and Kombar (*USQV2* and *USQV12*) were detected. Progeny isolates harbouring either *USQV2* or *USQV9* showed average disease reaction scores of 3.4 and 3.2, respectively, on Flagship. Isolates harbouring both *USQV2* and *USQV9* had a disease reaction score of 4.7 on Flagship. Progeny isolates harbouring either *USQV2* or *USQV12* showed average disease reaction scores of 3.8 and 4.4 on Kombar, respectively. Isolates harbouring both QTL associated with Kombar had an average disease reaction score of 5.8 on Kombar. QTL accumulation curves observed for the two QTL associated with the virulence on Kombar and two QTL associated with Flagship revealed that progeny isolates harbouring both QTL from each cultivar had a positive significant (*p* = 0.05) correlation with increased disease reaction scores on the respective cultivar (Figure 4).

Four QTL associated with the qualitative trait of having either net-like or spot-like symptoms were identified (*USQNB5.1, USQNB5.2, USQNB11* and *USQNB12*), with LOD values ranging from 3.0 to 3.9. The phenotypic variance explained by these QTL ranged from 7 to 9%.

### Candidate and Effectors Genes

The QTL regions (20 kb flanking regions on both side of the peak marker) were aligned with both reference genome annotations. Sixty-eight candidate genes were detected for five of the nine QTL regions (Supplementary Table S3). No candidate genes were found for the other four QTL. Out of 68 candidate genes, 12 genes were effector candidate genes with a score > 0.8 estimated by EffectorP and gene expression profile (Ismail and Able, 2016, 2017). Effector PTTW11_06577 associated with QTL *USQV5* is a known protein (G27; XP_003303420) that is expressed during net-form net blotch disease of barley (Ismail and Able, 2016, 2017). This effector gene was also reported to be associated with thioredoxin (PTTW11_06577; PF00085; Finn et al., 2016). Candidate gene PTTW11_06585 was also associated with QTL *USQV5* and was found to be an effector gene (G154; XP_003301637) by gene expression profiling. This gene is associated with the peptidase A4 family (PTTW11_06585; PF01828; Finn et al., 2016). The candidate gene PTMSG1_09710 found in *USQNB11* QTL region was responsible for Dolichol-phosphate mannosyltransferase production (PTMSG1_09710; PF00535). The candidate gene PTMSG1_10204 located within the region of QTL *USQV12* was associated with glycoside hydrolase family 45 proteins (PTMSG1_10204; PF02015). Only four predicted effector genes PTTW11_06577, PTTW11_06585, PTMSG1_09710 and PTMSG1_10204 had known protein domains according to the protein family database pfam (Finn et al., 2016). The other eight predicted effector genes were identified as hypothetical proteins.

## Discussion

To the author’s knowledge, this is the first study to use a hybrid population of *Pyrenophora teres* f. *teres* and *Pyrenophora teres* f. *maculata* in a QTL analysis study. Recent identification of an increasing number of hybrids in barley fields indicates the importance of understanding the virulence patterns of hybrid isolates (Turo et al., 2021). The results of this study give an insight into the virulence profile of hybrid isolates with respect to their parental isolates and provide useful information about barley-*P. teres* pathosystem.

Using a hybrid mapping population in this study enabled the development of a high-density genetic map consisting of 1,432 non-redundant markers with an average distance of 1.268 cM between flanking markers. In comparison, genetic maps of *Ptt/Ptt* or *Ptm/Ptm* bi-parental mapping population studies only had between 118 and 733 polymorphic markers (Weiland et al., 1999; Beattie et al., 2007; Lai et al., 2007; Shjerve et al., 2014; Carlsen et al., 2017; Koladia et al., 2017; Martin et al., 2020) thus suggesting that hybrid populations are more polymorphic. Deploying a hybrid mapping population enabled the detection of QTL present in both *Ptt* and *Ptm* genomes and allowed identification of genomic regions associated with the development of leaf symptoms caused by *Ptt* and *Ptm*.

Similar to Martin et al. (2020), three QTL, *USQV2, USQV9* and *USQV12*, identified in this study were associated with the virulence in more than one barley cultivar. These QTL could be associated with either a common protein responsible for the virulence in all the barley cultivars or they could be closely linked to multiple regions associated with multiple proteins responsible for individual cultivars. However, identification of common QTL regions (*USQV2, USQV9* and *USQV12*) responsible for the virulence of more than one cultivar in the current study confirms that some genomic regions are less host specific compared to unique QTL regions which were responsible for virulence on only one barley cultivar.

Most of the QTL identified in the current study are unique and novel. To date, seven bi-parental mapping studies for *Ptt* and *Ptm*, and one genome-wide association mapping study for *Ptt* have been conducted using different barley cultivars to detect genomic regions associated with avirulence/virulence of *P. teres* (Weiland et al., 1999; Beattie et al., 2007; Lai et al., 2007; Shjerve et al., 2014; Carlsen et al., 2017; Koladia et al., 2017; Martin et al., 2020). Three of the cultivars used in the present study, Fleet, Kombar and Prior, were also used in a genome-wide association mapping study conducted with Australian *P. teres* isolates (Martin et al., 2020). The study was conducted using the DArTseq™ marker system. Fourteen different genomic regions associated with virulence of *Ptt* were detected across 20 phenotyped barley cultivars. Some of these identified genomic regions were confirmed by QTL analysis of two bi-parental mapping populations, NB029/HRS09122 and NB029/NB085. The genomic regions associated with Kombar and Prior in the current study and the aforementioned study (Martin et al., 2020) were located in different regions of the *P. teres* genome. The QTL associated with Kombar and Prior virulence in the current study was contributed by the *Ptm* parent, while the QTL identified in the previous study (Martin et al., 2020) were contributed by *Ptt*. This confirms that different isolates or different pathotypes of *Ptt* and *Ptm* have different effectors to infect the same barley cultivar. Existence of a diverse spectrum of pathotypes of *P. teres* isolates (Boungab et al., 2012; Oğuz and Karakaya, 2017) also suggests the diversity of effectors secreted by individual *P. teres* isolates to infect the host.

Shjerve et al. (2014) used a cross between two Californian *Ptt* isolates (15A and 6A) with different virulence reactions to Rika and Kombar. They detected two virulence loci, *VK1* and *VK2*, for the Kombar cultivar and another two loci, *VR1* and *VR2*, for the Rika cultivar (Shjerve et al., 2014). The QTL, *VK1* and *VK2* were not detected in our study although the Kombar phenotype was also used. Similarly, two bi-parental mapping studies (Koladia et al., 2017; Martin et al., 2020) which both used the cultivar Beecher did not detect the same QTL regions. One study used a cross between an isolate from Denmark and one from the United States and the other a cross between two Australian *Ptt* isolates. The authors suggested that genomic regions controlling the virulence of the same barley cultivar may not be conserved among geographically distant isolates (Martin et al., 2020).

One of the aims of this study was to identify the genomic regions associated with the net blotch leaf symptoms. However, most of the progeny isolates showed spot-like disease symptoms and only 13 of the progeny isolates could be clearly identified as having net-like symptoms. Similar observation was made for field collected hybrids which also all showed spot-like disease symptoms (Campbell et al., 2002; Mclean et al., 2014; Turo et al., 2021). Spot- and net-form symptoms are impossible to differentiate at the lower infection rates and thus, some of these progeny isolates could have been miss-classified as spot-form instead of net-form. Although four genomic regions associated with leaf symptoms were detected in this study, further studies using multiple-population QTL analysis are needed to verify these QTL regions. The infection process of *Ptt* and *Ptm* is reported to be different between the two forms with *Ptt* having a necrotrophic life cycle while *Ptm* initially appears to develop as a biotroph and later transforming into a necrotroph (Lightfoot and Able, 2010). Most of the effectors secreted by plant pathogens show species specificity due to the co-evolution of their hosts (Sonah et al., 2016; Kim et al., 2019). Even though *Ptt* and *Ptm* belong to the same species, since they are from different forms it would be plausible that *Ptt* and *Ptm* could secrete different sets of effectors. Furthermore, the infection processes and development of disease symptoms of the pathogen have been proposed to be complex events (Lightfoot and Able, 2010; Liu et al., 2011), indicating that, there could be a number of genes and effectors associated with *P. teres* infection and disease development on barley.

A study conducted with SNP markers using the *Ptt* population BB25/FGOH04Ptt-21 reported nine unique QTL responsible for the virulence on eight different barley cultivars (Koladia et al., 2017). One of the QTL, *PttBee2,* which was detected in the Koladia et al. (2017) study to be responsible for the virulence on Beecher was co-localised with leaf symptom QTL *USQNB5.2* in our study. Two QTL detected by Martin et al. (2020) using GWAS, QTL11 and QTL12 on chromosome 5 and identified in a bi-parental mapping population in the same study were also co-located with QTL, *PttBee_5* and *USQNB5.2* in the current study. Co-localization of the leaf symptom QTL with those for virulence suggests that some genomic regions responsible for virulence in *P. teres* may have effects on determining the leaf symptoms of the pathogen or that these genes could be closely linked to each other. QTL *VK2*, associated with the virulence on Kombar detected on chromosome 2 (Shjerve et al., 2014), and QTL *PttSki_5*, associated with the virulence on Skiff (Koladia et al., 2017), were also located close to QTL *USQV2* and *USQV5*, which were associated with virulence on Kombar and Fleet, respectively, in the current study.

Three hybrid isolates (*Pop37*_41, *Pop37*_63 and *Pop37*_339) were virulent on all 20 cultivars tested including some of the currently used net blotch-resistance cultivars. A detailed examination of the genotypic data of isolates *Pop37*_41, *Pop37*_63 and *Pop37*_339 indicated that *Pop37*_41 harbours three (*USQV2, USQV5* and *USQV8*), *Pop37*_63 harbours two (*USQV5* and *USQV8*) and *Pop37*_339 harbours four (*USQV2, USQV8, USQV9* and *USQV12*) QTL associated with virulence. Phenotypic assessment of the Fathom cultivar with these three isolates revealed that even though both parental isolates (NB63 and HSR07033) were avirulent on Fathom, hybrid isolates showed increased virulence on Fathom. As these hybrid isolates have both *Ptt* and *Ptm* virulence genes, they would be valuable to breeders for testing barley cultivars for both spot-form net blotch and net-form net blotch resistance at the same time.

The QTL accumulation curves observed for the disease reaction scores of Kombar and Flagship suggest that multiple QTL can significantly increase the disease severity and indicate the potential devastating damage hybrid progenies could have on the barley industry in the absence of suitable resistant barley cultivars. Most current Australian cultivars are moderately susceptible to susceptible (MSS) to spot-form (GRDC, 2020). Hence, to develop suitable cultivar with resistance to *P. teres* hybrids, barley breeders will need to incorporate both net-form and spot-form resistance QTL into one cultivar.

This is the first study to attempt QTL mapping of disease symptoms of the net blotches. The large number of different QTL, including unique QTL, identified in this study point to a complex interaction between *P. teres* and its barley host. This study has demonstrated that hybrid isolates are viable and can accumulate virulence genes of both forms. Thus, hybrid populations can accelerate the evolution of the pathogen and overcome the host resistance more rapidly than *Ptt*x*Ptt* or *Ptm*x*Ptm* populations. This suggests that it is essential to introgress barley resistance genes of both forms of *P. teres* into new barley germplasm.

## Data Availability Statement

The raw data supporting the conclusions of this article will be made available by the authors, without undue reservation.

## Author Contributions

BD and AM designed the experiment. BD conducted the experiment, analysed the data, and wrote and revised the manuscript. AM, LS, and NV revised the manuscript. All authors contributed to the article and approved the submitted version.

## Funding

This project was partly funded by the Grains Research and Development Corporation (GRDC), Australia, grant number DAQ00187.

## Conflict of Interest

The authors declare that the research was conducted in the absence of any commercial or financial relationships that could be construed as a potential conflict of interest.

## Publisher’s Note

All claims expressed in this article are solely those of the authors and do not necessarily represent those of their affiliated organizations, or those of the publisher, the editors and the reviewers. Any product that may be evaluated in this article, or claim that may be made by its manufacturer, is not guaranteed or endorsed by the publisher.
