# Supplementary material for: Using a Hybrid Mapping Population to Identify Genomic Regions of Pyrenophora teres Associated With Virulence
Source: Front Plant Sci. 2022 Jun 23;13:925107. doi: 10.3389/fpls.2022.925107 (PMC9260246; doi:10.3389/fpls.2022.925107)
Supplement: Supplementary file 1 [file Data_Sheet_1.pdf]

Supplementary Table 1. Results for ten *Pyrenophora teres* progeny isolates and parent isolates of *Pop37* showing disease reaction scores on 20 barley cultivars

| Isolates | *Pop37_* 41 | *Pop37_* 48 | *Pop37_* 52 | *Pop37_* 63 | *Pop37_* 74 | *Pop37_* 237 | *Pop37_* 245 | *Pop37_* 249 | *Pop37_* 339 | *Pop37_* 362 | NB63 | HRS 07033 |
|---|---|---|---|---|---|---|---|---|---|---|---|---|
| Clho 5791* | 8.0 | 7.0 | 8.0 | 8.0 | 7.0 | 6.5 | 8.0 | 8.0 | 9.0 | 8.5 | 1.0 | 6.0 |
| Dampier* | 7.0 | 6.0 | 8.0 | 8.5 | 7.0 | 7.0 | 9.0 | 7.0 | 8.5 | 7.0 | 8.0 | 3.5 |
| Flagship* | 8.0 | 6.0 | 7.5 | 7.5 | 6.5 | 6.0 | 6.0 | 6.0 | 8.0 | 8.0 | 6.5 | 3.5 |
| Fleet* | 7.0 | 6.0 | 8.0 | 7.5 | 6.0 | 6.5 | 7.0 | 8.0 | 8.0 | 8.0 | 5.0 | 2.0 |
| Gairdner* | 8.0 | 7.0 | 8.5 | 8.5 | 7.0 | 8.0 | 8.0 | 7.5 | 9.0 | 8.5 | 8.0 | 5.0 |
| Grimmett* | 6.0 | 7.0 | 8.0 | 7.5 | 8.0 | 6.5 | 7.0 | 6.5 | 9.0 | 8.0 | 7.5 | 3.5 |
| Kombar* | 6.0 | 7.0 | 7.0 | 8.5 | 6.5 | 7.5 | 7.0 | 7.0 | 8.0 | 8.0 | 6.5 | 7.0 |
| Prior* | 6.0 | 8.0 | 8.5 | 8.5 | - | 7.0 | 8.5 | 7.5 | 8.5 | 8.5 | 9.5 | 5.0 |
| Beecher | 6.2 | 6.0 | 4.0 | 6.0 | 2.3 | 5.0 | 4.3 | 3.5 | 6.0 | 4.5 | 4.0 | 3.0 |
| Clho 11458 | 6.0 | 5.8 | 5.0 | 6.5 | 2.5 | 5.5 | 4.8 | 4.8 | 8.5 | 6.0 | 5.5 | 5.8 |
| Compass | 7.0 | 2.8 | 4.3 | 6.5 | 2.0 | 4.8 | 5.0 | 3.5 | 7.0 | 5.0 | 6.5 | 6.8 |
| Fathom | 7.0 | 4.0 | 6.3 | 7.5 | 0.5 | 4.8 | 3.8 | 4.0 | 5.3 | 4.5 | 1.0 | 1.0 |
| Harbin | 6.0 | 5.8 | 4.5 | 5.2 | 1.0 | 5.5 | 2.5 | 3.0 | 5.5 | 4.8 | 7.0 | 5.3 |
| Keel | 8.0 | 4.3 | 5.8 | 7.5 | 4.0 | 4.8 | 4.8 | 5.3 | 6.0 | 5.0 | 8.3 | 4.0 |
| Navigator | 7.8 | 3.5 | 4.8 | 8.0 | 0.5 | 4.5 | 1.3 | 3.8 | 7.5 | 4.5 | 5.8 | 4.0 |
| RGT Planet | 8.0 | 4.3 | 5.8 | 7.5 | 1.8 | 5.0 | 4.0 | 4.8 | 7.8 | 4.8 | 3.5 | 6.3 |
| Rosalind | 8.0 | 5.5 | 6.0 | 6.0 | 2.5 | 5.5 | 4.0 | 4.0 | 8.5 | 5.0 | 4.5 | 6.3 |
| Schooner | 7.0 | 4.5 | 3.0 | 6.3 | 2.0 | 4.5 | 2.8 | 3.3 | 8.0 | 4.8 | 8.0 | 5.0 |
| Spartacus CL | 8.5 | 5.0 | 5.0 | 8.5 | 2.0 | 4.5 | 4.0 | 3.0 | 7.8 | 5.5 | 6.5 | 6.3 |
| Vlamingh | 6.5 | 5.5 | 5.0 | 6.3 | 1.3 | 5.0 | 3.3 | 4.0 | 8.5 | 5.5 | 5.3 | 6.0 |

* Eight barley cultivars used in QTL analysis.

Supplementary Table S2. Marker order of the genetic map of *Pop37* and the corresponding marker position of the two reference genomes; W1-1 and SG1

| *Pop37*_Chr01 | | | W1-1 | | SG1 | |
|---|---|---|---|---|---|---|
| Marker order | Marker ID | cM | Marker order | Base pair | Market order | Base pair |
| 1 | 36350590 | 0.6006 | 1 | 411511 | 1 | 173836 |
| 2 | 36348241 | 0.9083 | 2 | 418770 | 2 | 181292 |
| 3 | 36346381 | 4.4752 | 3 | 421039 | 3 | 183562 |
| 4 | 36348174 | 5.1101 | 4 | 426173 | 4 | 188692 |
| 5 | 36346923 | 7.0158 | 9 | 691885 | 9 | 302244 |
| 6 | 28946156 | 9.0284 | 6 | 740484 | 6 | 350269 |
| 7 | 28947716 | 11.0468 | 10 | 950620 | 10 | 574331 |
| 8 | 36350940 | 13.7935 | 7 | 974975 | 11 | 595968 |
| 9 | 36345988 | 15.1096 | 12 | 997285 | 7 | 596033 |
| 10 | 36346005 | 17.6434 | 13 | 1007502 | 12 | 618218 |
| 11 | 36349470 | 19.6735 | 14 | 1020669 | 14 | 641623 |
| 12 | 36347906 | 21.2463 | 15 | 1046383 | 15 | 667353 |
| 13 | 36349009 | 21.5638 | 16 | 1049138 | 16 | 670136 |
| 14 | 28947304 | 22.4436 | 19 | 1111357 | 18 | 731745 |
| 15 | 28948967 | 23.0251 | 20 | 1192023 | 19 | 731810 |

| | | | | | | |
|---|---|---|---|---|---|---|
| 16 | 36349901 | 28.2615 | 21 | 1198439 | 20 | 812414 |
| 17 | 28948849 | 28.8412 | 22 | 1209462 | 21 | 818822 |
| 18 | 36351550 | 29.4844 | 23 | 1225957 | 22 | 829620 |
| 19 | 36350228 | 34.386 | 25 | 1272246 | 23 | 846089 |
| 20 | 36350818 | 34.6881 | 26 | 1294707 | 24 | 892358 |
| 21 | 28947570 | 35.5578 | 30 | 1329420 | 25 | 892358 |
| 22 | 36349690 | 36.43 | 31 | 1334999 | 27 | 910592 |
| 23 | 28946600 | 39.1111 | 33 | 1366387 | 29 | 940863 |
| 24 | 28946431 | 39.4276 | 34 | 1378930 | 30 | 940928 |
| 25 | 36350781 | 42.243 | 35 | 1387434 | 31 | 946497 |
| 26 | 36346614 | 43.1895 | 36 | 1431642 | 33 | 977882 |
| 27 | 36348886 | 43.5285 | 38 | 1539703 | 34 | 990428 |
| 28 | 28947277 | 45.1847 | 39 | 1548277 | 35 | 998927 |
| 29 | 28945144 | 45.4963 | 40 | 1559727 | 36 | 1043140 |
| 30 | 36349862 | 45.7887 | 41 | 1574641 | 37 | 1086327 |
| 31 | 36348761 | 47.5646 | 42 | 1587421 | 38 | 1151046 |
| 32 | 36346711 | 47.88 | 43 | 1599594 | 39 | 1159635 |
| 33 | 36346060 | 48.8206 | 44 | 1632148 | 40 | 1171085 |
| 34 | 28950112 | 49.4037 | 45 | 1635557 | 42 | 1198468 |

| | | | | | | |
|---|---|---|---|---|---|---|
| 35 | 28947631 | 49.6919 | 48 | 1708275 | 43 | 1210704 |
| 36 | 28945654 | 52.2804 | 49 | 1715021 | 44 | 1243256 |
| 37 | 36349698 | 59.4079 | 50 | 1743107 | 45 | 1246666 |
| 38 | 28949848 | 60.0104 | 47 | 1757376 | 48 | 1319365 |
| 39 | 36349464 | 60.6039 | 46 | 1763532 | 49 | 1326117 |
| 40 | 28947928 | 61.5186 | 51 | 1838680 | 50 | 1354121 |
| 41 | 28947665 | 61.8235 | 52 | 1851358 | 47 | 1368394 |
| 42 | 28948613 | 62.7085 | 53 | 1939879 | 46 | 1374549 |
| 43 | 36347564 | 63.0181 | 54 | 2135723 | 51 | 1449648 |
| 44 | 36348123 | 63.3249 | 55 | 2153712 | 52 | 1462318 |
| 45 | 36348303 | 66.3487 | 56 | 2177813 | 53 | 1550897 |
| 46 | 36346308 | 66.6832 | 57 | 2187759 | 54 | 1634989 |
| 47 | 28946720 | 67.2856 | 58 | 2202265 | 55 | 1652984 |
| 48 | 36350777 | 67.6124 | 59 | 2230958 | 56 | 1677062 |
| 49 | 36348805 | 67.9329 | 60 | 2231536 | 57 | 1766266 |
| 50 | 36346671 | 74.053 | 61 | 2273753 | 58 | 1780690 |
| 51 | 36350728 | 74.6628 | 62 | 2318176 | 59 | 1809391 |
| 52 | 28947958 | 76.7048 | 63 | 2337452 | 60 | 1809969 |
| 53 | 36348509 | 76.9921 | 66 | 2405233 | 61 | 1852080 |

| | | | | | | |
|---|---|---|---|---|---|---|
| 54 | 36350564 | 77.5669 | 67 | 2425603 | 62 | 1872591 |
| 55 | 28947354 | 77.8542 | 69 | 2465787 | 63 | 1891705 |
| 56 | 28946536 | 78.7239 | 70 | 2469467 | 64 | 1959411 |
| 57 | 28947997 | 80.182 | 73 | 2506394 | 66 | 1959411 |
| 58 | 36348278 | 81.0671 | 71 | 2506580 | 67 | 1979787 |
| 59 | 28947683 | 81.4004 | 75 | 2726335 | 68 | 2020315 |
| 60 | 36347905 | 83.4354 | 76 | 2802176 | 69 | 2020315 |
| 61 | 36348578 | 83.7549 | 77 | 2814354 | 70 | 2024000 |
| 62 | 28946967 | 84.3939 | 78 | 2826644 | 72 | 2060948 |
| 63 | 36348917 | 87.0592 | 79 | 2847888 | 73 | 2060948 |
| 64 | 28948372 | 87.644 | 80 | 2849439 | 71 | 2061134 |
| 65 | 36346710 | 88.2671 | 81 | 2857252 | 75 | 2107857 |
| 66 | 36349426 | 88.9102 | 82 | 2865471 | 77 | 2198030 |
| 67 | 36346757 | 90.8529 | 83 | 2876240 | 78 | 2210321 |
| 68 | 36348337 | 91.1645 | 84 | 2909402 | 79 | 2231615 |
| 69 | 28946626 | 91.829 | 85 | 2915942 | 80 | 2233158 |
| 70 | 36346303 | 92.8291 | 86 | 2925445 | 81 | 2240939 |
| 71 | 28945792 | 93.134 | #N/A | | 82 | 2249135 |
| 72 | 36349585 | 93.4325 | #N/A | | 83 | 2259967 |

| | | | | | | |
|---|---|---|---|---|---|---|
| 73 | 36349586 | 93.731 | #N/A | | 84 | 2296816 |
| 74 | 28949757 | 96.0651 | #N/A | | 85 | 2303279 |
| 75 | 28946368 | 99.1083 | #N/A | | 86 | 2312772 |
| 76 | 28945704 | 99.4179 | #N/A | | #N/A | |
| 77 | 36349628 | 100.9244 | #N/A | | #N/A | |
| 78 | 36348772 | 101.5759 | #N/A | | #N/A | |
| 79 | 36346518 | 102.219 | #N/A | | #N/A | |
| 80 | 36348777 | 102.5149 | #N/A | | #N/A | |
| 81 | 36348544 | 102.8099 | #N/A | | #N/A | |
| 82 | 28948128 | 106.1381 | #N/A | | #N/A | |
| 83 | 36346553 | 107.3541 | #N/A | | #N/A | |
| 84 | 28948925 | 107.6456 | #N/A | | #N/A | |
| 85 | 28948453 | 107.9398 | #N/A | | #N/A | |
| 86 | 28948149 | 107.99 | #N/A | | #N/A | |
| *Pop37_* Chr02 | | | W1-1 | | SG1 | |
| Marker order | Marker ID | cM | Marker order | Base pair | Marker order | Base pair |
| 1 | 28947153 | 0.2976 | 3 | 280936 | 2 | 125561 |
| 2 | 36348929 | 0.5917 | 4 | 284271 | 3 | 125730 |

| | | | | | | |
|---|---|---|---|---|---|---|
| 3 | 28945265 | 0.8885 | 1 | 317210 | 4 | 129288 |
| 4 | 28947085 | 4.7537 | 5 | 337069 | 1 | 162250 |
| 5 | 28946283 | 5.3617 | 7 | 346503 | 5 | 182038 |
| 6 | 28950221 | 5.9887 | 8 | 358195 | 7 | 192984 |
| 7 | 36346791 | 8.4446 | 10 | 359343 | 8 | 204769 |
| 8 | 36350689 | 9.0417 | 11 | 360380 | 10 | 205912 |
| 9 | 36347802 | 9.3358 | 9 | 743099 | 11 | 206970 |
| 10 | 36351297 | 10.2734 | 13 | 1408024 | 9 | 416116 |
| 11 | 36345801 | 10.5909 | 15 | 1457316 | 14 | 512990 |
| 12 | 36346079 | 12.7661 | 16 | 1523474 | 13 | 575919 |
| 13 | 36347767 | 13.3493 | 17 | 1542208 | 15 | 625155 |
| 14 | 28947926 | 17.3694 | 18 | 1546108 | 16 | 691329 |
| 15 | 36348200 | 19.3936 | 19 | 1558514 | 17 | 710130 |
| 16 | 28949827 | 19.6843 | 21 | 1575337 | 18 | 714026 |
| 17 | 36347686 | 19.9901 | 20 | 1699790 | 19 | 724954 |
| 18 | 36350559 | 20.9104 | 22 | 1738535 | 21 | 741922 |
| 19 | 36349602 | 21.2063 | 25 | 1745663 | 20 | 742763 |
| 20 | 36346294 | 21.5084 | 26 | 1745728 | 22 | 781470 |
| 21 | 28948170 | 23.7181 | 23 | 1753677 | 25 | 788622 |

| | | | | | | |
|---|---|---|---|---|---|---|
| 22 | 36350656 | 24.6736 | 28 | 1766167 | 26 | 788687 |
| 23 | 36349936 | 24.9871 | 24 | 1767074 | 23 | 796527 |
| 24 | 36348713 | 25.2948 | 27 | 1784625 | 24 | 809943 |
| 25 | 36347999 | 25.5987 | 29 | 1807136 | 27 | 828089 |
| 26 | 36346070 | 25.9142 | 30 | 1868620 | 29 | 850672 |
| 27 | 36350035 | 26.8667 | 31 | 1900673 | 30 | 911273 |
| 28 | 28947535 | 30.0866 | 32 | 1906896 | 31 | 943302 |
| 29 | 36348633 | 31.4807 | 33 | 1913846 | 32 | 949526 |
| 30 | 36349740 | 33.3624 | 35 | 1953511 | 33 | 956501 |
| 31 | 28948448 | 34.2321 | 37 | 2001297 | 35 | 996142 |
| 32 | 36348938 | 34.5228 | 36 | 2005683 | 37 | 1045131 |
| 33 | 36349782 | 35.6691 | 38 | 2114007 | 36 | 1049517 |
| 34 | 28947429 | 36.2422 | 43 | 2114007 | 38 | 1140232 |
| 35 | 36351140 | 38.8233 | 41 | 2126839 | 43 | 1140232 |
| 36 | 28947789 | 41.2568 | 42 | 2144424 | 41 | 1153109 |
| 37 | 36345731 | 44.6289 | 39 | 2149202 | 42 | 1170713 |
| 38 | 36349865 | 45.6324 | 44 | 2149202 | 39 | 1175638 |
| 39 | 36348404 | 48.4045 | 40 | 2181946 | 44 | 1175638 |
| 40 | 28947848 | 49.2617 | 45 | 2238961 | 40 | 1208398 |

| | | | | | | |
|---|---|---|---|---|---|---|
| 41 | 28948996 | 52 | 46 | 2239738 | 45 | 1265769 |
| 42 | 36350117 | 53.6785 | 47 | 2258613 | 46 | 1266546 |
| 43 | 28946286 | 54.0175 | 48 | 2325602 | 47 | 1285736 |
| 44 | 28946301 | 58.5342 | 50 | 2388838 | 48 | 1352609 |
| 45 | 28947010 | 58.8721 | 51 | 2485443 | 50 | 1418031 |
| 46 | 36350122 | 59.6129 | 52 | 2507344 | 51 | 1514464 |
| 47 | 36351343 | 62.1958 | 55 | 2547471 | 52 | 1536364 |
| 48 | 28947318 | 62.4865 | 53 | 2584949 | 54 | 1571181 |
| 49 | 36346720 | 64.571 | 56 | 2629640 | 55 | 1576654 |
| 50 | 28947826 | 66.9538 | 58 | 2667501 | 53 | 1614089 |
| 51 | 36350069 | 67.9003 | 59 | 2668973 | 56 | 1658657 |
| 52 | 36347668 | 69.1624 | 57 | 2670777 | 58 | 1696461 |
| 53 | 36348780 | 70.0771 | 60 | 2725996 | 59 | 1697861 |
| 54 | 36346222 | 71.0118 | 61 | 2730966 | 60 | 1754684 |
| 55 | 28948625 | 72.4828 | 62 | 2747624 | 61 | 1759694 |
| 56 | 36348198 | 75.18 | 63 | 2790313 | 62 | 1776361 |
| 57 | 28947583 | 75.4777 | 64 | 2830947 | 63 | 1819092 |
| 58 | 36348147 | 75.7798 | 65 | 2888165 | 64 | 1859731 |
| 59 | 36346387 | 79.7394 | 67 | 2906333 | 65 | 1918084 |

| | | | | | | |
|---|---|---|---|---|---|---|
| 60 | 28948472 | 80.8924 | 68 | 2910208 | 67 | 1935218 |
| 61 | 28948344 | 81.5293 | 66 | 2912044 | 68 | 1938717 |
| 62 | 28945168 | 84.513 | 69 | 2984626 | 66 | 1940554 |
| 63 | 28946469 | 84.8198 | 70 | 2996453 | 69 | 2013104 |
| 64 | 36347514 | 86.6443 | 71 | 2997719 | 70 | 2024899 |
| 65 | 36348153 | 87.8906 | 72 | 3004141 | 71 | 2026165 |
| 66 | 36345763 | 88.2002 | 73 | 3004141 | 72 | 2032597 |
| 67 | 36350002 | 88.7902 | 75 | 3029889 | 73 | 2032597 |
| 68 | 36350512 | 91.5285 | 76 | 3068500 | 74 | 2050741 |
| 69 | 36348631 | 93.6446 | 77 | 3068500 | 75 | 2058336 |
| 70 | 28946700 | 93.9336 | 78 | 3084971 | 76 | 2096968 |
| 71 | 28948174 | 94.2312 | 79 | 3119385 | 77 | 2096968 |
| 72 | 28946852 | 94.5361 | 80 | 3196781 | 78 | 2113439 |
| 73 | 28946072 | 94.8373 | 81 | 3524805 | 79 | 2147877 |
| 74 | 28945449 | 96.0646 | 82 | 3558798 | 80 | 2225353 |
| 75 | 36349026 | 96.3732 | 83 | 3588931 | 81 | 2627877 |
| 76 | 28949961 | 96.68 | 84 | 3600707 | 82 | 2661884 |
| 77 | 28949960 | 97.8532 | 85 | 3611361 | 83 | 2692041 |
| 78 | 36349742 | 98.7356 | 86 | 3642207 | 84 | 2703793 |

| | | | | | | |
|---|---|---|---|---|---|---|
| 79 | 28947553 | 100.1981 | 87 | 3683334 | 85 | 2714473 |
| 80 | 28948113 | 100.4922 | 88 | 3687987 | 86 | 2745344 |
| 81 | 36349872 | 101.3746 | 89 | 3720531 | 87 | 2786491 |
| 82 | 28948245 | 106.0257 | 90 | 3732294 | 88 | 2791138 |
| 83 | 28950092 | 106.8928 | 92 | 3779722 | 89 | 2823654 |
| 84 | 36348646 | 107.8246 | 91 | 3784489 | 90 | 2835434 |
| 85 | 28947216 | 108.4596 | 93 | 3797497 | 92 | 2882941 |
| 86 | 36348348 | 109.3633 | 102 | 3824585 | 91 | 2887707 |
| 87 | 36347826 | 109.6583 | 100 | 3843731 | 93 | 2900657 |
| 88 | 36349508 | 110.5486 | 101 | 3858796 | 102 | 2926223 |
| 89 | 28945656 | 111.1917 | 95 | 3880610 | 101 | 2960754 |
| 90 | 36346910 | 112.1352 | 94 | 3890886 | 95 | 2982345 |
| 91 | 28945831 | 112.4419 | 96 | 3890886 | 94 | 2992594 |
| 92 | 36347592 | 113.4522 | 99 | 3898369 | 96 | 2992594 |
| 93 | 36346109 | 117.4474 | 98 | 3909480 | 99 | 3000088 |
| 94 | 36347670 | 118.3705 | 97 | 3916870 | 98 | 3011202 |
| 95 | 28948650 | 118.684 | 103 | 3958419 | 97 | 3018592 |
| 96 | 36346883 | 119.323 | 104 | 4035616 | 103 | 3060021 |
| 97 | 36349000 | 119.6252 | 110 | 4035616 | 104 | 3137308 |

| | | | | | | |
|---|---|---|---|---|---|---|
| 98 | 28948703 | 121.1594 | 109 | 4035681 | 110 | 3137308 |
| 99 | 41805310 | 122.7472 | 105 | 4091367 | 105 | 3193111 |
| 100 | 28946282 | 123.9745 | 108 | 4091367 | 108 | 3193111 |
| 101 | 36349752 | 125.1341 | 106 | 4112186 | 106 | 3348900 |
| 102 | 28947900 | 132.9331 | 113 | 4169857 | 107 | 3363998 |
| 103 | 28949382 | 136.8687 | 112 | 4176511 | 113 | 3406671 |
| 104 | 36349418 | 137.4937 | 111 | 4181342 | 112 | 3413331 |
| 105 | 36347987 | 137.8024 | 114 | 4181342 | 111 | 3418130 |
| 106 | 41805581 | 138.0906 | 115 | 4223591 | 114 | 3418130 |
| 107 | 36349816 | 138.3763 | 117 | 4251254 | 115 | 3460024 |
| 108 | 28948349 | 140.9182 | 118 | 4251254 | 117 | 3486843 |
| 109 | 28947468 | 141.2515 | 119 | 4278442 | 118 | 3486843 |
| 110 | 100303497 | 143.8597 | 121 | 4278507 | 119 | 3513899 |
| 111 | 28945689 | 144.7944 | 120 | 4329776 | 121 | 3513964 |
| 112 | 28946336 | 145.9851 | 122 | 4382494 | 120 | 3557417 |
| 113 | 36349373 | 146.9376 | 123 | 4495923 | 123 | 3721573 |
| 114 | 36349973 | 149.4795 | 124 | 4496550 | 124 | 3722200 |
| 115 | 28947980 | 150.9505 | 125 | 4512707 | 125 | 3738383 |
| 116 | 28949869 | 151.266 | 126 | 4545576 | 126 | 3771352 |

| | | | | | | |
|---|---|---|---|---|---|---|
| 117 | 36347525 | 151.6096 | 127 | 4567816 | 127 | 3793563 |
| 118 | 36346821 | 152.2327 | 128 | 4597394 | 128 | 3821996 |
| 119 | 28947560 | 152.8333 | 129 | 4646821 | 129 | 3884435 |
| 120 | 36348717 | 154.072 | 130 | 4662018 | 130 | 3899623 |
| 121 | 36345798 | 154.9839 | 135 | 4668142 | 135 | 3905743 |
| 122 | 28945270 | 164.7145 | 133 | 4683101 | 133 | 3920713 |
| 123 | 36348296 | 165.0185 | 132 | 4696164 | 131 | 3928239 |
| 124 | 36346829 | 166.567 | 131 | 4696448 | 132 | 3928239 |
| 125 | 28948167 | 166.862 | 136 | 4866741 | 136 | 4071006 |
| 126 | 28945286 | 168.0284 | 134 | 4869891 | 134 | 4074158 |
| 127 | 28947869 | 168.3182 | 137 | 4909395 | 137 | 4113687 |
| 128 | 28949006 | 168.9047 | 138 | 4922090 | 138 | 4126272 |
| 129 | 28948054 | 169.7795 | 139 | 4957903 | 139 | 4162245 |
| 130 | 28946961 | 170.4045 | 140 | 4957903 | 140 | 4162245 |
| 131 | 36351189 | 170.722 | 141 | 4963245 | 141 | 4167577 |
| 132 | 28949400 | 171.0161 | 142 | 4975852 | 142 | 4180029 |
| 133 | 28946813 | 172.2586 | 143 | 4980474 | 143 | 4184651 |
| 134 | 36346835 | 172.5843 | 145 | 5003764 | 145 | 4208099 |
| 135 | 36346897 | 173.2173 | 144 | 5018828 | 144 | 4223168 |

| | | | | | | |
|---|---|---|---|---|---|---|
| 136 | 28947381 | 176.8163 | 146 | 5042270 | 148 | 4272804 |
| 137 | 36347980 | 177.1268 | 148 | 5068491 | 150 | 4314045 |
| 138 | 28945871 | 180.443 | 149 | 5099713 | 151 | 4354315 |
| 139 | 28947280 | 180.7938 | 150 | 5109848 | 152 | 4374395 |
| 140 | 36349024 | 182.0599 | 151 | 5147358 | 153 | 4384257 |
| 141 | 36348243 | 182.9529 | 152 | 5167364 | #N/A | |
| 142 | 28946241 | 183.8621 | 153 | 5177243 | #N/A | |
| 143 | 36349515 | 185.7498 | 154 | 5205986 | #N/A | |
| 144 | 36349547 | 186.673 | #N/A | | #N/A | |
| 145 | 28948440 | 187.5739 | #N/A | | #N/A | |
| 146 | 28946983 | 187.8761 | #N/A | | #N/A | |
| 147 | 36350430 | 189.7878 | #N/A | | #N/A | |
| 148 | 36346924 | 190.8296 | #N/A | | #N/A | |
| 149 | 28949187 | 191.4547 | #N/A | | #N/A | |
| 150 | 28947953 | 193.4966 | #N/A | | #N/A | |
| 151 | 28949396 | 197.0009 | #N/A | | #N/A | |
| 152 | 28949880 | 198.7357 | #N/A | | #N/A | |
| 153 | 36350509 | 200.092 | #N/A | | #N/A | |
| 154 | 28946936 | 200.093 | #N/A | | #N/A | |

| *Pop37_* Chr03 | | | W1-1 | | SG1 | |
|---|---|---|---|---|---|---|
| Marker order | Marker ID | cM | Marker order | Base pair | Marker order | Base pair |
| 1 | 36349905 | 0.304 | 3 | 149605 | 1 | 137557 |
| 2 | 36349027 | 0.6061 | 2 | 892055 | 5 | 162916 |
| 3 | 36349032 | 1.5098 | 4 | 1098678 | 6 | 192636 |
| 4 | 28949140 | 1.8119 | 5 | 1559717 | 7 | 206984 |
| 5 | 36350224 | 2.7584 | 6 | 1589628 | 11 | 250412 |
| 6 | 36346345 | 3.3776 | 7 | 1604004 | 9 | 260631 |
| 7 | 28946232 | 4.3065 | 11 | 1650412 | 10 | 267213 |
| 8 | 36349309 | 4.9219 | 9 | 1660628 | 12 | 267213 |
| 9 | 28947142 | 6.4801 | 10 | 1667224 | 13 | 270578 |
| 10 | 36349693 | 7.1253 | 12 | 1667224 | 15 | 288035 |
| 11 | 36347966 | 8.7387 | 15 | 1687975 | 14 | 298555 |
| 12 | 36349692 | 9.3819 | 14 | 1698505 | 17 | 326490 |
| 13 | 36348628 | 10.3721 | 16 | 1704527 | 19 | 387418 |
| 14 | 41804653 | 11.9351 | 17 | 1726459 | 18 | 400386 |
| 15 | 36347908 | 12.2266 | 19 | 1794205 | 20 | 403103 |
| 16 | 28948401 | 14.3686 | 18 | 1807169 | 21 | 443534 |

| | | | | | | |
|---|---|---|---|---|---|---|
| 17 | 28946463 | 20.5731 | 20 | 1809890 | 22 | 475794 |
| 18 | 28945560 | 20.8743 | 22 | 1880484 | 23 | 475794 |
| 19 | 36351333 | 21.1719 | 23 | 1880484 | 24 | 520324 |
| 20 | 36347663 | 22.7448 | 24 | 1925537 | 26 | 583155 |
| 21 | 28945773 | 23.0553 | 26 | 1988242 | 25 | 611026 |
| 22 | 28945370 | 23.7005 | 25 | 2016098 | 27 | 635611 |
| 23 | 28945371 | 24.9469 | 27 | 2040663 | 28 | 654086 |
| 24 | 28948357 | 27.2947 | 28 | 2059179 | 30 | 672483 |
| 25 | 36347534 | 27.6052 | 30 | 2077575 | 29 | 688974 |
| 26 | 36351278 | 31.3389 | 29 | 2094066 | 31 | 710849 |
| 27 | 28946351 | 31.6504 | 31 | 2115939 | 32 | 718179 |
| 28 | 36346834 | 36.1936 | 32 | 2123272 | 33 | 747505 |
| 29 | 36348269 | 36.5081 | 33 | 2152599 | 36 | 850192 |
| 30 | 36345903 | 38.6834 | 36 | 2237422 | 34 | 873722 |
| 31 | 36350705 | 39.5632 | 34 | 2261191 | 35 | 876768 |
| 32 | 28949718 | 40.7296 | 35 | 2264239 | 37 | 882826 |
| 33 | 36348077 | 42.8848 | 37 | 2270297 | 38 | 893547 |
| 34 | 36346908 | 43.1887 | 38 | 2281024 | 39 | 914942 |
| 35 | 36348020 | 43.8278 | 39 | 2302434 | 40 | 924907 |

| | | | | | | |
|---|---|---|---|---|---|---|
| 36 | 28946216 | 44.1462 | 40 | 2330021 | 41 | 1184513 |
| 37 | 28948381 | 47.048 | 41 | 2337700 | 43 | 1223188 |
| 38 | 28948143 | 48.2043 | 43 | 2519106 | 44 | 1243483 |
| 39 | 36349573 | 48.784 | 44 | 2539338 | 45 | 1248790 |
| 40 | 28948941 | 51.6719 | 45 | 2544648 | 42 | 1250645 |
| 41 | 36348552 | 52.6621 | 42 | 2546504 | 46 | 1304242 |
| 42 | 36346072 | 52.9921 | 46 | 2599878 | 47 | 1329433 |
| 43 | 36348026 | 55.5179 | 47 | 2625034 | 48 | 1350995 |
| 44 | 36351564 | 55.8053 | 48 | 2646613 | 50 | 1358305 |
| 45 | 28948734 | 57.2425 | 49 | 2672012 | 49 | 1376219 |
| 46 | 36349395 | 58.9723 | 51 | 2673875 | 51 | 1378075 |
| 47 | 28946975 | 60.1286 | 52 | 2700983 | 52 | 1405177 |
| 48 | 28946845 | 60.4201 | 53 | 2702770 | 53 | 1406958 |
| 49 | 28948008 | 62.6297 | 56 | 2729873 | 56 | 1427298 |
| 50 | 36345901 | 63.2791 | 54 | 2755999 | 54 | 1450540 |
| 51 | 36348487 | 63.9481 | 57 | 2787476 | 57 | 1482032 |
| 52 | 36350650 | 66.8359 | 58 | 2912654 | 58 | 1507102 |
| 53 | 36348718 | 67.4073 | 61 | 2924391 | 61 | 1518768 |
| 54 | 28947634 | 67.7095 | 60 | 2928552 | 60 | 1522930 |

| | | | | | | |
|---|---|---|---|---|---|---|
| 55 | 36351066 | 68.0191 | 59 | 2937879 | 59 | 1532236 |
| 56 | 36346322 | 72.1583 | 62 | 2937880 | 62 | 1532237 |
| 57 | 28945730 | 76.2017 | 63 | 2962716 | 63 | 1556829 |
| 58 | 36350106 | 76.8059 | 64 | 3020573 | 64 | 1614757 |
| 59 | 28946551 | 77.4194 | 65 | 3076085 | 65 | 1670036 |
| 60 | 36349835 | 79.3251 | 66 | 3102260 | 66 | 1702500 |
| 61 | 36345797 | 79.6509 | 67 | 3127208 | 67 | 1727440 |
| 62 | 36345983 | 82.8501 | 68 | 3135042 | 68 | 1735268 |
| 63 | 36346338 | 84.9347 | 69 | 3135042 | 69 | 1735268 |
| 64 | 36349884 | 85.222 | 70 | 3141284 | 70 | 1741497 |
| 65 | 28947635 | 86.0917 | 71 | 3157822 | 71 | 1758028 |
| 66 | 28947690 | 87.8571 | 73 | 3186100 | 72 | 2168989 |
| 67 | 28949352 | 88.4419 | 72 | 3417238 | 75 | 2168989 |
| 68 | 36348081 | 88.7478 | 75 | 3417238 | 74 | 2185736 |
| 69 | 28946559 | 92.4814 | 74 | 3433968 | 77 | 2237312 |
| 70 | 28949368 | 93.0875 | 77 | 3485530 | 76 | 2251644 |
| 71 | 28947992 | 95.9029 | 76 | 3499868 | 78 | 2290922 |
| 72 | 36348722 | 96.8585 | 78 | 3539429 | 79 | 2349655 |
| 73 | 36348210 | 98.7055 | 79 | 3598142 | 80 | 2409173 |

| | | | | | | |
|---|---|---|---|---|---|---|
| 74 | 28949964 | 101.1997 | 80 | 3657667 | 82 | 2421006 |
| 75 | 36348721 | 105.6846 | 82 | 3669484 | 81 | 2431464 |
| 76 | 36346471 | 106.3361 | 81 | 3679937 | 83 | 2553167 |
| 77 | 28945385 | 107.6022 | 83 | 3801654 | 84 | 2612158 |
| 78 | 28946532 | 108.4693 | 84 | 3891615 | 85 | 2673817 |
| 79 | 28945917 | 114.0479 | 85 | 3953289 | 86 | 2697711 |
| 80 | 28948731 | 114.9654 | 86 | 3977184 | 88 | 2730988 |
| 81 | 36348206 | 115.2819 | 88 | 4010412 | 89 | 2763932 |
| 82 | 28945152 | 118.9247 | 89 | 4043295 | 90 | 2803347 |
| 83 | 28947059 | 119.2146 | 90 | 4082701 | 91 | 3075436 |
| 84 | 28948232 | 121.4035 | 91 | 4338379 | 92 | 3075775 |
| 85 | 28948210 | 122.0285 | 92 | 4338714 | 93 | 3135960 |
| 86 | 28947666 | 122.9135 | 93 | 4398983 | 94 | 3140049 |
| 87 | 36346713 | 123.2085 | 94 | 4403127 | 95 | 3177003 |
| 88 | 28947623 | 124.3648 | 95 | 4440274 | 96 | 3182195 |
| 89 | 28949971 | 124.9412 | 96 | 4445467 | 97 | 3185744 |
| 90 | 28948077 | 125.2294 | 97 | 4449013 | 99 | 3192509 |
| 91 | 28948267 | 125.5235 | 99 | 4455802 | 98 | 3192608 |
| 92 | 36348871 | 127.7614 | 98 | 4455900 | 100 | 3204920 |

| | | | | | | |
|---|---|---|---|---|---|---|
| 93 | 36346530 | 128.0769 | 100 | 4468209 | 101 | 3207294 |
| 94 | 28947605 | 130.7109 | 101 | 4470583 | 102 | 3224663 |
| 95 | 28946578 | 132.2404 | 102 | 4487954 | 103 | 3263082 |
| 96 | 36349591 | 133.1959 | 103 | 4526327 | 104 | 3270631 |
| 97 | 28945863 | 133.8249 | 104 | 4533911 | 106 | 3296740 |
| 98 | 36346329 | 134.7369 | 105 | 4539369 | 107 | 3296740 |
| 99 | 28947945 | 137.3632 | 106 | 4560083 | 108 | 3323311 |
| 100 | 28949939 | 137.669 | 107 | 4560083 | 109 | 3347157 |
| 101 | 28947616 | 141.0277 | 108 | 4586639 | 111 | 3368355 |
| 102 | 36349629 | 144.6928 | 109 | 4610437 | 110 | 3368420 |
| 103 | 36346531 | 145.6103 | 111 | 4631687 | 113 | 3385892 |
| 104 | 28945559 | 147.7789 | 110 | 4631752 | 112 | 3386220 |
| 105 | 28945700 | 148.4039 | 113 | 4649075 | 114 | 3398072 |
| 106 | 28946468 | 148.7125 | 112 | 4649403 | 115 | 3421327 |
| 107 | 36350549 | 151.6004 | 114 | 4662225 | 120 | 3468219 |
| 108 | 28945166 | 157.3954 | 115 | 4685328 | 3 | 3517117 |
| 109 | 36350851 | 158.6041 | #N/A | | #N/A | |
| 110 | 28947301 | 158.9466 | #N/A | | #N/A | |
| 111 | 28946652 | 160.2758 | #N/A | | #N/A | |

| | | | | | | |
|---|---|---|---|---|---|---|
| 112 | 36347738 | 160.6125 | #N/A | | #N/A | |
| 113 | 36346203 | 162.6136 | #N/A | | #N/A | |
| 114 | 28947884 | 167.271 | #N/A | | #N/A | |
| 115 | 36346097 | 167.5844 | #N/A | | #N/A | |
| 116 | 28945757 | 168.8005 | #N/A | | #N/A | |
| 117 | 28948041 | 169.1017 | #N/A | | #N/A | |
| 118 | 36349207 | 169.402 | #N/A | | #N/A | |
| 119 | 28949965 | 169.7041 | #N/A | | #N/A | |
| 120 | 36345919 | 169.7042 | #N/A | | #N/A | |
| *Pop37_*Chr04 | | | W1-1 | | SG1 | |
| Marker order | Marker ID | cM | Marker order | Base pair | Marker order | Base pair |
| 1 | 36348166 | 0.3268 | 1 | 44565 | 2 | 39178 |
| 2 | 36346339 | 1.2388 | 3 | 68653 | 1 | 59946 |
| 3 | 36348686 | 4.5975 | 4 | 72477 | 3 | 81467 |
| 4 | 36350916 | 7.6223 | 5 | 82261 | 4 | 85234 |
| 5 | 36347830 | 12.5515 | 7 | 142625 | 5 | 95127 |
| 6 | 28946183 | 12.8439 | 6 | 142900 | 6 | 155645 |
| 7 | 28946122 | 15.3227 | 9 | 172367 | 9 | 185295 |

| | | | | | | |
|---|---|---|---|---|---|---|
| 8 | 36347960 | 15.6304 | 8 | 172930 | 8 | 185857 |
| 9 | 36348723 | 16.2101 | 10 | 197730 | 10 | 210660 |
| 10 | 36351214 | 21.6367 | 12 | 221542 | 12 | 237637 |
| 11 | 28949332 | 22.5773 | 13 | 227434 | 13 | 244300 |
| 12 | 28945424 | 24.1452 | 14 | 235638 | 14 | 252525 |
| 13 | 36345921 | 24.4558 | 15 | 254368 | 15 | 271257 |
| 14 | 28947335 | 27.3623 | 16 | 274739 | 16 | 291452 |
| 15 | 36346457 | 29.6219 | 17 | 315305 | 17 | 313967 |
| 16 | 28945630 | 30.1999 | 18 | 365858 | 18 | 364327 |
| 17 | 36348274 | 33.0066 | 19 | 377528 | 19 | 375988 |
| 18 | 36345658 | 34.253 | 20 | 390882 | 20 | 389375 |
| 19 | 36348666 | 35.4126 | 21 | 433286 | 21 | 431868 |
| 20 | 36350277 | 37.4605 | 22 | 437647 | 22 | 436225 |
| 21 | 28946946 | 37.759 | 23 | 441521 | 23 | 440100 |
| 22 | 36346430 | 39.2432 | 24 | 481340 | 24 | 479751 |
| 23 | 28948993 | 42.6969 | 25 | 522310 | 25 | 522984 |
| 24 | 28947014 | 43.5641 | 26 | 546627 | 26 | 547293 |
| 25 | 28949507 | 44.1438 | 27 | 566043 | 27 | 566720 |
| 26 | 28946681 | 44.7355 | 28 | 581129 | 28 | 581822 |

| | | | | | | |
|---|---|---|---|---|---|---|
| 27 | 36347557 | 45.3238 | 29 | 598204 | 29 | 599079 |
| 28 | 28947385 | 46.761 | 30 | 621502 | 30 | 622388 |
| 29 | 36351295 | 48.2023 | 35 | 719696 | 35 | 720760 |
| 30 | 28946629 | 48.777 | 31 | 939848 | 31 | 1015391 |
| 31 | 28947682 | 49.678 | 36 | 946822 | 36 | 1089206 |
| 32 | 36349861 | 49.9783 | 34 | 1029710 | 34 | 1172085 |
| 33 | 28948732 | 50.5632 | 32 | 1071050 | 32 | 1213392 |
| 34 | 28949896 | 50.8573 | 33 | 1092947 | 33 | 1233766 |
| 35 | 28948099 | 52.1194 | 37 | 1106375 | 37 | 1247280 |
| 36 | 36346761 | 52.7688 | 38 | 1144507 | 38 | 1284085 |
| 37 | 28945579 | 57.2826 | 39 | 1178014 | 40 | 1400214 |
| 38 | 36348232 | 58.2261 | 40 | 1205584 | 41 | 1427165 |
| 39 | 28947260 | 58.516 | 41 | 1232498 | 42 | 1457623 |
| 40 | 28946672 | 58.8041 | 42 | 1262949 | 43 | 1496590 |
| 41 | 28945650 | 59.9505 | 43 | 1301905 | 44 | 1537933 |
| 42 | 28946034 | 61.2246 | 44 | 1343236 | 45 | 1563235 |
| 43 | 28945254 | 63.1487 | 45 | 1368538 | 47 | 1615281 |
| 44 | 36348510 | 66.6529 | 47 | 1420746 | 48 | 1615346 |
| 45 | 28948881 | 67.8508 | 48 | 1420811 | 49 | 1627734 |

| | | | | | | |
|---|---|---|---|---|---|---|
| 46 | 36352001 | 68.1466 | 49 | 1433461 | 53 | 1644246 |
| 47 | 36349894 | 68.446 | 53 | 1449973 | 55 | 1644246 |
| 48 | 28945680 | 69.0614 | 55 | 1449973 | 54 | 2005057 |
| 49 | 28946177 | 70.3316 | 54 | 1555668 | 56 | 2034817 |
| 50 | 36348707 | 71.2781 | 56 | 1585458 | 50 | 2035408 |
| 51 | 28946264 | 71.5829 | 50 | 1586049 | 52 | 2077479 |
| 52 | 28948468 | 71.8711 | 52 | 1684307 | 51 | 2077544 |
| 53 | 28947134 | 72.1635 | 51 | 1684372 | 57 | 2091843 |
| 54 | 36352286 | 72.482 | 57 | 1698100 | 59 | 2091843 |
| 55 | 36347561 | 72.812 | 59 | 1698100 | 58 | 2118049 |
| 56 | 28947017 | 73.116 | 58 | 1724096 | 60 | 2129703 |
| 57 | 28948368 | 73.4285 | 60 | 1735720 | 61 | 2138876 |
| 58 | 28945319 | 74.0516 | 61 | 1745005 | 63 | 2176154 |
| 59 | 36347493 | 76.9534 | 63 | 1782292 | 62 | 2191530 |
| 60 | 28945881 | 77.5348 | 62 | 1797701 | 64 | 2197645 |
| 61 | 28946046 | 82.2687 | 64 | 1803813 | 65 | 2230676 |
| 62 | 36348837 | 82.9016 | 65 | 1836804 | 66 | 2244788 |
| 63 | 36348583 | 83.9017 | 66 | 1850870 | 67 | 2260332 |
| 64 | 28948865 | 85.1599 | 67 | 1866408 | 68 | 2266385 |

| | | | | | | |
|---|---|---|---|---|---|---|
| 65 | 36347653 | 86.0346 | 68 | 1872458 | 70 | 2342439 |
| 66 | 36348648 | 88.2099 | 70 | 1947235 | 69 | 2370794 |
| 67 | 28946904 | 88.8291 | 69 | 1975608 | 72 | 2388207 |
| 68 | 28947601 | 90.2663 | 72 | 1993013 | 71 | 2393239 |
| 69 | 28947463 | 92.0908 | 71 | 1998045 | 73 | 2424226 |
| 70 | 36346050 | 92.3994 | 73 | 2029044 | 74 | 2433177 |
| 71 | 36348088 | 94.2753 | 74 | 2037995 | 76 | 2455309 |
| 72 | 36349292 | 95.4877 | 76 | 2060148 | 75 | 2510663 |
| 73 | 36347983 | 95.7944 | 75 | 2144835 | 78 | 2537231 |
| 74 | 36346902 | 96.6954 | 78 | 2171182 | 77 | 2560879 |
| 75 | 28949236 | 97.3146 | 79 | 2204237 | 79 | 2570156 |
| 76 | 36346900 | 98.597 | 80 | 2264846 | 80 | 2630693 |
| 77 | 36346894 | 98.9075 | 81 | 2282917 | 81 | 2648516 |
| 78 | 28947123 | 99.1991 | 82 | 2330005 | 82 | 2695541 |
| 79 | 28946864 | 100.7524 | 83 | 2343732 | 83 | 2708943 |
| 80 | 36346476 | 101.4147 | 84 | 2377469 | 84 | 2742686 |
| 81 | 36350266 | 106.7811 | 85 | 2502837 | 85 | 2791506 |
| 82 | 36349587 | 107.1133 | 86 | 2512844 | 86 | 2801519 |
| 83 | 36348366 | 108.0939 | 89 | 2531845 | 88 | 2820396 |

| | | | | | | |
|---|---|---|---|---|---|---|
| 84 | 28949247 | 109.2435 | 88 | 2531910 | 87 | 2844462 |
| 85 | 28946814 | 109.5292 | 87 | 2555560 | 94 | 2852384 |
| 86 | 28949404 | 109.8166 | 94 | 2563487 | 95 | 2852824 |
| 87 | 28946453 | 112.3504 | 95 | 2563926 | 90 | 2855434 |
| 88 | 36346846 | 112.6894 | 90 | 2566528 | 93 | 2869684 |
| 89 | 36346819 | 113.3473 | 93 | 2580770 | 92 | 2873051 |
| 90 | 36346868 | 115.2291 | 92 | 2584269 | 96 | 2899195 |
| 91 | 36348300 | 116.3689 | 96 | 2610526 | 91 | 2901431 |
| 92 | 36351582 | 117.5739 | 91 | 2612759 | 97 | 2913946 |
| 93 | 28945843 | 117.8845 | 97 | 2625303 | 100 | 2941425 |
| 94 | 28948408 | 118.195 | 98 | 2642603 | 99 | 2954991 |
| 95 | 28946717 | 119.7728 | 100 | 2652945 | 102 | 3029138 |
| 96 | 28946587 | 120.1018 | 99 | 2666508 | 104 | 3042219 |
| 97 | 36347775 | 120.7939 | 102 | 2740587 | 105 | 3057293 |
| 98 | 28947019 | 121.4518 | 103 | 2753668 | 106 | 3064310 |
| 99 | 28946118 | 122.3778 | 105 | 2768772 | 107 | 3101988 |
| 100 | 28947314 | 125.8719 | 106 | 2777302 | 108 | 3115282 |
| 101 | 36349322 | 126.4533 | 107 | 2815003 | 109 | 3134844 |
| 102 | 36350679 | 127.3998 | 108 | 2828306 | 110 | 3142488 |

| | | | | | | |
|---|---|---|---|---|---|---|
| 103 | 28947734 | 128.7424 | 110 | 2855139 | 111 | 3153008 |
| 104 | 36346451 | 129.6571 | 111 | 2865677 | 114 | 3154307 |
| 105 | 28947210 | 130.2335 | 114 | 2866934 | 112 | 3172936 |
| 106 | 28948542 | 131.9485 | 112 | 2885588 | 113 | 3172936 |
| 107 | 28947625 | 132.2479 | 113 | 2885588 | #N/A | |
| 108 | 36349677 | 132.871 | #N/A | | #N/A | |
| 109 | 36348118 | 133.496 | #N/A | | #N/A | |
| 110 | 36346331 | 133.8125 | #N/A | | #N/A | |
| 111 | 36346433 | 134.123 | #N/A | | #N/A | |
| 112 | 36347845 | 135.721 | #N/A | | #N/A | |
| 113 | 28947201 | 136.4155 | #N/A | | #N/A | |
| 114 | 36346879 | 136.4156 | #N/A | | #N/A | |
| *Pop37_* Chr05 | | | W1-1 | | SG1 | |
| Marker order | Marker ID | cM | Marker order | Base pair | Marker order | Base pair |
| 1 | 28946097 | 0.2985 | 4 | 256820 | 4 | 224725 |
| 2 | 28947600 | 1.2361 | 3 | 283618 | 3 | 237821 |
| 3 | 36345879 | 1.8534 | 5 | 296106 | 5 | 248461 |
| 4 | 36347894 | 2.1433 | 6 | 302694 | 6 | 255079 |

| | | | | | | |
|---|---|---|---|---|---|---|
| 5 | 28949134 | 3.0104 | 7 | 307530 | 7 | 259926 |
| 6 | 36350089 | 4.2228 | 8 | 318123 | 8 | 270466 |
| 7 | 36346232 | 7.8356 | 10 | 440934 | 11 | 309976 |
| 8 | 36346350 | 11.6647 | 9 | 449886 | 13 | 311842 |
| 9 | 36350279 | 11.9651 | 11 | 526041 | 12 | 313831 |
| 10 | 28945886 | 12.8715 | 13 | 527908 | 14 | 339126 |
| 11 | 36346955 | 14.7242 | 12 | 529896 | 16 | 347324 |
| 12 | 28947163 | 15.0282 | 14 | 554487 | 15 | 369504 |
| 13 | 36348263 | 16.748 | 16 | 562706 | 17 | 406499 |
| 14 | 28948483 | 17.0362 | 15 | 594546 | 20 | 470158 |
| 15 | 36348562 | 18.5799 | 17 | 630067 | 25 | 504519 |
| 16 | 28945244 | 19.2129 | 20 | 700266 | 26 | 507256 |
| 17 | 28945890 | 21.7792 | 49 | 758240 | 24 | 520669 |
| 18 | 36350382 | 25.1482 | 21 | 925473 | 27 | 522433 |
| 19 | 36351483 | 25.4494 | 22 | 925752 | 28 | 530814 |
| 20 | 36346747 | 25.7515 | 24 | 941067 | 31 | 531558 |
| 21 | 36348992 | 26.0501 | 27 | 948596 | 23 | 537886 |
| 22 | 36350236 | 26.3459 | 23 | 958734 | 33 | 588897 |
| 23 | 28948839 | 28.8402 | #N/A | 987937 | 32 | 602290 |

| | | | | | | |
|---|---|---|---|---|---|---|
| 24 | 36348221 | 29.1577 | #N/A | 988266 | 49 | 626855 |
| 25 | 36346428 | 29.7926 | 33 | 990704 | #N/A | 635449 |
| 26 | 36351143 | 30.1091 | 32 | 1004247 | 30 | 663749 |
| 27 | 28945159 | 30.4187 | 63 | 1032493 | 29 | 678451 |
| 28 | 36348546 | 31.0854 | 30 | 1037367 | 36 | 708981 |
| 29 | 36346139 | 31.408 | 29 | 1052863 | 35 | 751206 |
| 30 | 36351222 | 31.703 | 36 | 1086026 | 34 | 796590 |
| 31 | 28947405 | 31.9988 | 35 | 1114747 | 37 | 797464 |
| 32 | 28946964 | 33.2569 | 34 | 1160777 | 38 | 852087 |
| 33 | 36346227 | 34.5602 | 37 | 1161558 | 39 | 985717 |
| 34 | 36348037 | 35.557 | 38 | 1229548 | 41 | 1028318 |
| 35 | 36348534 | 36.7995 | 39 | 1459682 | 43 | 1062109 |
| 36 | 28947188 | 37.3965 | 41 | 1527390 | 44 | 1124033 |
| 37 | 28946070 | 41.3202 | 43 | 1603621 | 45 | 1140442 |
| 38 | 28948427 | 46.2494 | 44 | 1657828 | 46 | 1195810 |
| 39 | 36348163 | 46.5427 | 45 | 1674365 | 47 | 1252372 |
| 40 | 28945776 | 47.1525 | 46 | 1760263 | 51 | 1333822 |
| 41 | 28946329 | 48.682 | 47 | 1826941 | 52 | 1333822 |
| 42 | 36351741 | 49.8621 | 54 | 1905727 | 55 | 1364907 |

| | | | | | | |
|---|---|---|---|---|---|---|
| 43 | 28949137 | 54.1148 | 51 | 1950384 | 57 | 1416743 |
| 44 | 36345649 | 54.4115 | 52 | 1950384 | 59 | 1425041 |
| 45 | 36349474 | 55.2712 | 55 | 1993119 | 56 | 1427456 |
| 46 | 36349917 | 55.8493 | 56 | 2027138 | 61 | 1454203 |
| 47 | 28946656 | 69.0772 | 61 | 2052267 | #N/A | 1493675 |
| 48 | 28950033 | 72.8501 | 62 | 2133316 | 62 | 1513935 |
| 49 | 36351675 | 82.1984 | 64 | 2168056 | 64 | 1548577 |
| 50 | 36351057 | 84.0627 | 65 | 2174517 | 65 | 1555040 |
| 51 | 36349490 | 84.3832 | 67 | 2229846 | 67 | 1610441 |
| 52 | 28949126 | 86.7874 | 66 | 2248958 | 66 | 1628608 |
| 53 | 36346814 | 87.1059 | 70 | 2291947 | 70 | 1671649 |
| 54 | 28945161 | 91.3093 | 72 | 2311485 | 72 | 1691128 |
| 55 | 28948051 | 91.6209 | 73 | 2317219 | 73 | 1696844 |
| 56 | 28945521 | 92.266 | 74 | 2331749 | 74 | 1711362 |
| 57 | 36348259 | 92.5664 | 71 | 2331965 | 71 | 1711578 |
| 58 | 36348413 | 93.1914 | 75 | 2341512 | 75 | 1721134 |
| 59 | 36346047 | 95.085 | 76 | 2393259 | 77 | 1775079 |
| 60 | 36346058 | 96.8557 | 77 | 2395396 | 80 | 1824761 |
| 61 | 28947055 | 99.4668 | 78 | 2401437 | 79 | 1829714 |

| | | | | | | |
|---|---|---|---|---|---|---|
| 62 | 28948480 | 100.3815 | 80 | 2446362 | 81 | 1873683 |
| 63 | 28945441 | 100.6911 | 79 | 2451316 | 82 | 1901317 |
| 64 | 28948354 | 100.9978 | 81 | 2495247 | 83 | 1903654 |
| 65 | 28948792 | 103.8133 | 82 | 2522683 | 84 | 1939092 |
| 66 | 36347807 | 104.1248 | 83 | 2525020 | 86 | 1979660 |
| 67 | 36347737 | 104.4216 | 84 | 2560433 | 87 | 2023038 |
| 68 | 28949082 | 105.0258 | 86 | 2600984 | 88 | 2037794 |
| 69 | 28948045 | 106.5648 | 87 | 2644308 | 89 | 2069882 |
| 70 | 36346768 | 108.5395 | 88 | 2659080 | 90 | 2075930 |
| 71 | 28946943 | 108.8674 | 89 | 2691216 | 91 | 2104412 |
| 72 | 28948240 | 109.1572 | 90 | 2697274 | 92 | 2149014 |
| 73 | 36349576 | 109.7543 | 91 | 2726338 | 93 | 2184712 |
| 74 | 36351363 | 110.6553 | 92 | 2770922 | 95 | 2279937 |
| 75 | 28946521 | 110.9511 | 93 | 2806612 | 94 | 2285045 |
| 76 | 36351587 | 111.8829 | 95 | 2903506 | 96 | 2296566 |
| 77 | 36346896 | 112.2208 | 94 | 2908613 | 99 | 2314033 |
| 78 | 36349314 | 114.9079 | 96 | 2920145 | 97 | 2317124 |
| 79 | 36346891 | 116.17 | 99 | 2937673 | 98 | 2317124 |
| 80 | 28947323 | 117.0346 | 97 | 2940780 | 103 | 2348520 |

| | | | | | | |
|---|---|---|---|---|---|---|
| 81 | 28946361 | 119.96 | 98 | 2940780 | 102 | 2378370 |
| 82 | 28945870 | 120.289 | 103 | 2972226 | 101 | 2396316 |
| 83 | 36349879 | 121.1741 | 102 | 3002077 | 100 | 2404916 |
| 84 | 28945399 | 122.0944 | 101 | 3019976 | 104 | 2416715 |
| 85 | 36349310 | 123.0945 | 100 | 3028592 | 105 | 2470773 |
| 86 | 28945502 | 123.7503 | 104 | 3040389 | 106 | 2512461 |
| 87 | 28948397 | 124.0552 | 105 | 3094483 | 107 | 2574226 |
| 88 | 28947132 | 124.935 | 106 | 3136364 | 109 | 2599192 |
| 89 | 28946994 | 126.1048 | 109 | 3232477 | 108 | 2878600 |
| 90 | 28948136 | 127.014 | 108 | 3461560 | 115 | 2891671 |
| 91 | 28946952 | 127.915 | 115 | 3474630 | 114 | 2907177 |
| 92 | 36348483 | 129.1771 | 114 | 3490159 | 110 | 2931117 |
| 93 | 36348662 | 133.889 | 110 | 3514596 | 113 | 2944250 |
| 94 | 28945588 | 134.2035 | 113 | 3527685 | 112 | 2981984 |
| 95 | 28948693 | 134.4917 | 112 | 3565412 | 116 | 2994162 |
| 96 | 36349730 | 136.51 | 116 | 3577591 | 111 | 2997249 |
| 97 | 36350745 | 136.8095 | 111 | 3580752 | 117 | 3025939 |
| 98 | 36350746 | 137.7589 | 117 | 3609441 | 118 | 3043251 |
| 99 | 36349050 | 140.1914 | 118 | 3626679 | 119 | 3047913 |

| | | | | | | |
|---|---|---|---|---|---|---|
| 100 | 36346777 | 141.7128 | 119 | 3631341 | 120 | 3063488 |
| 101 | 28947125 | 142.0407 | 120 | 3646873 | 121 | 3064403 |
| 102 | 28945620 | 142.3392 | 121 | 3647788 | 124 | 3078124 |
| 103 | 36345805 | 143.3632 | 124 | 3661729 | 122 | 3078458 |
| 104 | 36349803 | 147.1636 | 122 | 3662063 | 123 | 3086916 |
| 105 | 36346516 | 147.7946 | 125 | 3667045 | 126 | 3107425 |
| 106 | 28947252 | 150.2281 | 123 | 3670524 | 127 | 3136235 |
| 107 | 36346581 | 150.5257 | 126 | 3691016 | 128 | 3149319 |
| 108 | 28947799 | 151.1488 | 127 | 3719823 | 132 | 3161843 |
| 109 | 36347879 | 151.4663 | 128 | 3732926 | 130 | 3176027 |
| 110 | 36346683 | 151.7721 | 132 | 3745439 | 135 | 3212765 |
| 111 | 36349949 | 152.3638 | 130 | 3759621 | 134 | 3214011 |
| 112 | 100332252 | 152.6562 | 131 | 3777585 | 133 | 3259709 |
| 113 | 28948263 | 152.9799 | 135 | 3802272 | 136 | 3308405 |
| 114 | 36347878 | 153.3004 | 134 | 3803509 | 137 | 3352813 |
| 115 | 28947556 | 153.5869 | 133 | 3849330 | 138 | 3356241 |
| 116 | 28946245 | 154.7636 | 136 | 3985143 | 139 | 3400743 |
| 117 | 28946035 | 155.3571 | 137 | 4024670 | 140 | 3402563 |
| 118 | 28946951 | 155.9706 | 138 | 4028099 | 142 | 3451235 |

| | | | | | | |
|---|---|---|---|---|---|---|
| 119 | 36349588 | 156.3006 | 139 | 4072755 | 143 | 3484601 |
| 120 | 36347594 | 156.6212 | 140 | 4074577 | 147 | 3484601 |
| 121 | 36350725 | 157.5195 | 141 | 4099350 | 146 | 3485292 |
| 122 | 36348879 | 157.8262 | 142 | 4124938 | 145 | 3488468 |
| 123 | 36346691 | 158.132 | 143 | 4158103 | 144 | 3503878 |
| 124 | 28948410 | 158.7068 | 147 | 4158103 | 149 | 3519392 |
| 125 | 28946874 | 160.1357 | 146 | 4158808 | 148 | 3534044 |
| 126 | 36349945 | 161.9438 | 145 | 4161901 | 150 | 3534044 |
| 127 | 36346592 | 162.5535 | 144 | 4177324 | 151 | 3567852 |
| 128 | 28946240 | 163.755 | 148 | 4207551 | 152 | 3582971 |
| 129 | 36349287 | 164.3556 | 150 | 4207551 | 153 | 3635238 |
| 130 | 36350213 | 165.5791 | 151 | 4241364 | 154 | 3660673 |
| 131 | 36351724 | 166.6173 | 152 | 4341342 | 155 | 3675705 |
| 132 | 36345712 | 168.3014 | 153 | 4528823 | 156 | 3705144 |
| 133 | 36346113 | 169.2303 | 154 | 4554341 | 157 | 3713899 |
| 134 | 36349462 | 169.5262 | 155 | 4569376 | 158 | 3729840 |
| 135 | 36346686 | 171.3021 | 156 | 4598795 | 161 | 3752558 |
| 136 | 36347868 | 172.7393 | 157 | 4607583 | 160 | 3754143 |
| 137 | 28948978 | 173.0588 | 158 | 4623536 | 162 | 3768230 |

| | | | | | | |
|---|---|---|---|---|---|---|
| 138 | 36348524 | 174.7095 | 159 | 4626128 | 163 | 3786621 |
| 139 | 36350798 | 175.0191 | 161 | 4646135 | 164 | 3795772 |
| 140 | 28945586 | 177.6531 | 160 | 4647719 | 168 | 3852145 |
| 141 | 28945364 | 178.2397 | 162 | 4709550 | 165 | 3852282 |
| 142 | 36350672 | 178.5436 | 163 | 4727951 | 167 | 4162428 |
| 143 | 36350816 | 179.1553 | 164 | 4737000 | 166 | 4179717 |
| 144 | 28947659 | 179.4574 | 168 | 4793710 | 169 | 4220888 |
| 145 | 28945354 | 179.766 | 165 | 4793847 | 171 | 4237553 |
| 146 | 36348428 | 180.1145 | 167 | 4956981 | 172 | 4312116 |
| 147 | 36350817 | 182.9645 | 166 | 4974230 | 173 | 4329209 |
| 148 | 28946187 | 184.2306 | 169 | 5015459 | 180 | 4340150 |
| 149 | 36350589 | 184.8138 | 171 | 5032183 | 174 | 4348410 |
| 150 | 36349580 | 186.574 | 172 | 5049744 | 175 | 4359322 |
| 151 | 28948407 | 187.1554 | 173 | 5066839 | 179 | 4359322 |
| 152 | 36348623 | 187.4428 | 180 | 5077773 | 178 | 4360735 |
| 153 | 28946737 | 188.3049 | 174 | 5086039 | 176 | 4362659 |
| 154 | 28948883 | 188.8932 | 175 | 5096957 | 177 | 4362659 |
| 155 | 36348451 | 190.6744 | 179 | 5096957 | 181 | 4369396 |
| 156 | 28949113 | 191.5568 | 178 | 5098370 | 183 | 4378506 |

| | | | | | | |
|---|---|---|---|---|---|---|
| 157 | 36348120 | 192.1349 | 176 | 5100294 | 184 | 4411475 |
| 158 | 28948251 | 192.4388 | 177 | 5100294 | 186 | 4413961 |
| 159 | 36346053 | 193.3649 | 181 | 5107041 | 188 | 4467086 |
| 160 | 28946554 | 193.6697 | 183 | 5116230 | 189 | 4486932 |
| 161 | 36350840 | 193.9647 | 184 | 5149165 | 190 | 4530001 |
| 162 | 36350397 | 194.2597 | 186 | 5151650 | 191 | 4530001 |
| 163 | 28947041 | 194.8732 | 188 | 5515494 | 193 | 4538865 |
| 164 | 36348406 | 198.2526 | 189 | 5535344 | 194 | 4543437 |
| 165 | 28945706 | 198.552 | 192 | 5578635 | 196 | 4580442 |
| 166 | 28946440 | 198.8735 | 190 | 5579160 | 197 | 4595508 |
| 167 | 36351620 | 199.222 | 191 | 5579160 | 199 | 4608928 |
| 168 | 28947367 | 200.6064 | 193 | 5588048 | 198 | 4609564 |
| 169 | 28946009 | 200.9239 | 194 | 5592616 | 201 | 4612134 |
| 170 | 36350385 | 201.8115 | 195 | 5603364 | 200 | 4612135 |
| 171 | 36350631 | 203.8358 | 196 | 5629842 | 202 | 4618195 |
| 172 | 36348308 | 204.713 | 197 | 5644909 | 203 | 4636011 |
| 173 | 36348708 | 205.008 | 199 | 5658313 | 205 | 4644421 |
| 174 | 28945341 | 205.6234 | 198 | 5658947 | 204 | 4666990 |
| 175 | 36349941 | 206.2408 | 201 | 5661512 | 206 | 4707799 |

| | | | | | | |
|---|---|---|---|---|---|---|
| 176 | 36349980 | 206.5484 | 200 | 5661513 | 207 | 4721580 |
| 177 | 36349981 | 206.869 | 202 | 5667613 | 209 | 4792253 |
| 178 | 36349434 | 207.5424 | 203 | 5685635 | 210 | 4803470 |
| 179 | 28945671 | 208.5041 | 205 | 5694061 | 212 | 4853167 |
| 180 | 36349614 | 209.0838 | 204 | 5716646 | #N/A | |
| 181 | 36347759 | 209.3762 | 206 | 5757394 | #N/A | |
| 182 | 28950152 | 209.6848 | 207 | 5780239 | #N/A | |
| 183 | 36350501 | 210.0238 | 208 | 5824748 | #N/A | |
| 184 | 28947977 | 210.3605 | 209 | 5850941 | #N/A | |
| 185 | 28949056 | 211.2456 | 210 | 5863752 | #N/A | |
| 186 | 28947272 | 212.7845 | 212 | 6137080 | #N/A | |
| 187 | 36349583 | 213.1258 | #N/A | | #N/A | |
| 188 | 28945984 | 214.126 | #N/A | | #N/A | |
| 189 | 28947377 | 217.5689 | #N/A | | #N/A | |
| 190 | 36351267 | 217.8785 | #N/A | | #N/A | |
| 191 | 28947316 | 218.1834 | #N/A | | #N/A | |
| 192 | 28945644 | 218.4855 | #N/A | | #N/A | |
| 193 | 28948454 | 219.0844 | #N/A | | #N/A | |
| 194 | 28947358 | 220.3268 | #N/A | | #N/A | |

| | | | | | | |
|---|---|---|---|---|---|---|
| 195 | 36348512 | 224.0722 | #N/A | | #N/A | |
| 196 | 100281708 | 225.3185 | #N/A | | #N/A | |
| 197 | 36346753 | 226.2305 | #N/A | | #N/A | |
| 198 | 36351160 | 227.7461 | #N/A | | #N/A | |
| 199 | 36348410 | 229.0003 | #N/A | | #N/A | |
| 200 | 28945746 | 229.984 | #N/A | | #N/A | |
| 201 | 36346810 | 231.1927 | #N/A | | #N/A | |
| 202 | 36348217 | 232.0674 | #N/A | | #N/A | |
| 203 | 28949866 | 232.6506 | #N/A | | #N/A | |
| 204 | 36347639 | 233.5882 | #N/A | | #N/A | |
| 205 | 28945950 | 236.0206 | #N/A | | #N/A | |
| 206 | 36346156 | 236.7224 | #N/A | | #N/A | |
| 207 | 28948984 | 240.4561 | #N/A | | #N/A | |
| 208 | 28948268 | 246.2139 | #N/A | | #N/A | |
| 209 | 36347618 | 248.6623 | #N/A | | #N/A | |
| 210 | 28948057 | 251.031 | #N/A | | #N/A | |
| 211 | 36346914 | 254.3095 | #N/A | | #N/A | |
| 212 | 36350252 | 254.3096 | #N/A | | #N/A | |

| *Pop37_* Chr06 | | | W1-1 | | SG1 | |
|---|---|---|---|---|---|---|
| Marker order | Marker ID | cM | Marker order | Base pair | Marker order | Base pair |
| 1 | 28947322 | 0.304 | 1 | 36435 | 1 | 81983 |
| 2 | 36346717 | 0.6126 | 4 | 173298 | 4 | 97290 |
| 3 | 41805818 | 4.1328 | 5 | 184861 | 6 | 109709 |
| 4 | 36346529 | 7.8664 | 6 | 185736 | 9 | 120250 |
| 5 | 28945753 | 9.157 | 9 | 196338 | 8 | 132404 |
| 6 | 36345819 | 13.1389 | 8 | 208480 | 7 | 132833 |
| 7 | 28949784 | 16.2789 | 7 | 208910 | 11 | 193210 |
| 8 | 36350872 | 16.6361 | 11 | 269363 | 10 | 195160 |
| 9 | 28946061 | 22.0529 | 10 | 271316 | 12 | 207217 |
| 10 | 36348536 | 23.327 | 12 | 283392 | 13 | 213739 |
| 11 | 28946447 | 23.6273 | 13 | 289914 | 14 | 222842 |
| 12 | 36351294 | 23.925 | 14 | 320319 | 15 | 247386 |
| 13 | 28947511 | 27.127 | 15 | 344718 | 16 | 261066 |
| 14 | 28950076 | 29.9873 | 16 | 358403 | 17 | 266397 |
| 15 | 36349572 | 32.5291 | 17 | 363732 | 18 | 272036 |
| 16 | 28947767 | 33.6854 | 18 | 369154 | 19 | 281180 |

| | | | | | | |
|---|---|---|---|---|---|---|
| 17 | 28946871 | 34.2989 | 19 | 378330 | 20 | 285429 |
| 18 | 36345802 | 34.9125 | 20 | 382583 | 22 | 347511 |
| 19 | 28947346 | 35.2183 | 22 | 445282 | 23 | 357965 |
| 20 | 36346766 | 35.5269 | 23 | 455736 | 24 | 384880 |
| 21 | 36352236 | 41.122 | 24 | 482637 | 26 | 423808 |
| 22 | 28947286 | 41.411 | 27 | 586702 | 27 | 508578 |
| 23 | 28946688 | 41.7051 | 25 | 700912 | 25 | 585334 |
| 24 | 28945947 | 41.9967 | 28 | 767821 | 28 | 652232 |
| 25 | 36347649 | 43.7993 | 29 | 780541 | 29 | 664959 |
| 26 | 36345908 | 44.4302 | 30 | 880427 | 30 | 752809 |
| 27 | 36346016 | 46.0181 | 31 | 1346732 | 31 | 1118628 |
| 28 | 36348019 | 46.9245 | 32 | 1357243 | 32 | 1129152 |
| 29 | 28947935 | 49.2316 | 33 | 1393607 | 33 | 1165329 |
| 30 | 28945633 | 49.8047 | 34 | 1399552 | 35 | 1171268 |
| 31 | 28947382 | 51.8289 | 40 | 1423047 | 40 | 1194740 |
| 32 | 28946854 | 52.7219 | 41 | 1428713 | 41 | 1200395 |
| 33 | 36347958 | 53.0268 | 38 | 1430476 | 38 | 1202167 |
| 34 | 36348946 | 53.3494 | 37 | 1431001 | 37 | 1202692 |
| 35 | 36345685 | 56.2843 | 36 | 1443505 | 36 | 1215198 |

| | | | | | | |
|---|---|---|---|---|---|---|
| 36 | 36348379 | 56.621 | 39 | 1443505 | 39 | 1215198 |
| 37 | 36348261 | 57.3564 | 42 | 1458378 | 42 | 1230068 |
| 38 | 36346437 | 60.8369 | 43 | 1518752 | 43 | 1290358 |
| 39 | 36348378 | 63.57 | 44 | 1536875 | 44 | 1308846 |
| 40 | 28945304 | 65.6119 | 48 | 1558514 | 48 | 1330546 |
| 41 | 28948465 | 66.7682 | 47 | 1560246 | 47 | 1332253 |
| 42 | 28949628 | 72.6779 | 46 | 1571301 | 46 | 1343333 |
| 43 | 36350794 | 74.6206 | 49 | 1581117 | 45 | 1363004 |
| 44 | 36346447 | 75.6343 | 45 | 1591102 | #N/A | |
| 45 | 36348500 | 75.9569 | #N/A | | #N/A | |
| 46 | 36345676 | 78.4747 | #N/A | | #N/A | |
| 47 | 36348991 | 79.4008 | #N/A | | #N/A | |
| 48 | 36346730 | 79.7265 | #N/A | | #N/A | |
| 49 | 36346765 | 79.7266 | #N/A | | #N/A | |
| *Pop37_* Chr07 | | | W1-1 | | SG1 | |
| Marker order | Marker ID | cM | Marker order | Base pair | Marker order | Base pair |
| 1 | 28946604 | 0.3068 | 1 | 612111 | 1 | 192936 |
| 2 | 36349817 | 2.6895 | 2 | 612176 | 2 | 193001 |

| | | | | | | |
|---|---|---|---|---|---|---|
| 3 | 28949762 | 3.6271 | 4 | 626170 | 4 | 206978 |
| 4 | 28948134 | 6.9858 | 5 | 657312 | 7 | 265121 |
| 5 | 36346353 | 7.8814 | 9 | 728943 | 9 | 309397 |
| 6 | 28950108 | 10.9528 | 10 | 745063 | 10 | 325456 |
| 7 | 36346950 | 12.5256 | 11 | 750815 | 11 | 363720 |
| 8 | 28948096 | 13.1606 | 12 | 757968 | 12 | 386867 |
| 9 | 36351321 | 13.7876 | 13 | 813200 | 13 | 420148 |
| 10 | 36346770 | 15.6289 | 14 | 867415 | 14 | 474749 |
| 11 | 28945255 | 16.2224 | 15 | 901498 | 15 | 564421 |
| 12 | 28947338 | 17.0772 | 16 | 935352 | 17 | 598273 |
| 13 | 28947956 | 22.2788 | 17 | 935352 | 19 | 621342 |
| 14 | 36346952 | 22.5846 | 18 | 947506 | 20 | 650519 |
| 15 | 28948639 | 24.0556 | 19 | 958432 | 21 | 698273 |
| 16 | 28949724 | 24.3596 | 20 | 987645 | 23 | 759159 |
| 17 | 36346936 | 24.962 | 21 | 1035393 | 22 | 766565 |
| 18 | 36350393 | 25.5451 | 23 | 1106110 | 25 | 869151 |
| 19 | 28948409 | 26.1404 | 22 | 1113516 | 27 | 877710 |
| 20 | 28949415 | 30.6984 | 24 | 1206108 | 26 | 899982 |
| 21 | 36348590 | 36.5733 | 25 | 1216763 | 28 | 940019 |

| | | | | | | |
|---|---|---|---|---|---|---|
| 22 | 28946513 | 37.2042 | 27 | 1225313 | 30 | 1043363 |
| 23 | 28948749 | 42.0113 | 26 | 1247571 | 31 | 1066171 |
| 24 | 36346272 | 46.5693 | 28 | 1287851 | 32 | 1084798 |
| 25 | 36346926 | 47.8558 | 30 | 1390960 | 33 | 1111276 |
| 26 | 28949782 | 50.4304 | 31 | 1413762 | 34 | 1127346 |
| 27 | 28949127 | 52.7921 | 32 | 1432253 | 35 | 1160344 |
| 28 | 36349734 | 53.6931 | 33 | 1547585 | 36 | 1225554 |
| 29 | 36351745 | 55.1728 | 34 | 1563660 | 37 | 1241680 |
| 30 | 28947155 | 56.0399 | 35 | 1596720 | 38 | 1272349 |
| 31 | 36347545 | 56.6572 | 36 | 1661973 | 39 | 1300527 |
| 32 | 28946613 | 57.5833 | 37 | 1678132 | 40 | 1318554 |
| 33 | 36348394 | 57.8967 | 38 | 1708877 | 47 | 1358288 |
| 34 | 36346858 | 58.8523 | 39 | 1737112 | 48 | 1360080 |
| 35 | 36346878 | 61.2279 | 40 | 1755154 | 50 | 1384331 |
| 36 | 28948518 | 61.5153 | 47 | 1795112 | 41 | 1642547 |
| 37 | 36347665 | 61.8202 | 48 | 1796909 | 49 | 1780680 |
| 38 | 28948278 | 62.1438 | 50 | 1821250 | 42 | 1792382 |
| 39 | 36350843 | 62.7592 | 41 | 1950024 | 44 | 1822863 |
| 40 | 36347842 | 63.339 | 49 | 2328709 | 43 | 1824688 |

| | | | | | | |
|---|---|---|---|---|---|---|
| 41 | 28945985 | 63.6272 | 42 | 2340292 | 51 | 1858505 |
| 42 | 28948660 | 64.2103 | 45 | 2345384 | 52 | 1892931 |
| 43 | 28947444 | 64.5504 | 44 | 2371067 | 54 | 1906035 |
| 44 | 36348651 | 67.0797 | 43 | 2372892 | 53 | 1944298 |
| 45 | 36346323 | 67.7269 | 51 | 2406751 | 55 | 1950905 |
| 46 | 28948595 | 69.5739 | 52 | 2441127 | 56 | 1950907 |
| 47 | 28947326 | 71.7358 | 54 | 2454001 | 57 | 1962273 |
| 48 | 36346637 | 73.0606 | 53 | 2492166 | 58 | 2008484 |
| 49 | 41805640 | 73.3928 | 55 | 2498776 | 59 | 2042410 |
| 50 | 36346771 | 77.7081 | 56 | 2498778 | 60 | 2118522 |
| 51 | 28945849 | 81.4186 | 57 | 2510167 | 61 | 2123019 |
| 52 | 28946875 | 81.7119 | 58 | 2556386 | 63 | 2129292 |
| 53 | 28948928 | 82.0254 | 59 | 2590866 | 62 | 2137268 |
| 54 | 36345860 | 83.6337 | 60 | 2666924 | 68 | 2137273 |
| 55 | 36349791 | 83.9648 | 61 | 2671421 | 64 | 2154471 |
| 56 | 36349792 | 84.2725 | 63 | 2677694 | 67 | 2154471 |
| 57 | 28947469 | 84.559 | 62 | 2685655 | 66 | 2154536 |
| 58 | 28948434 | 85.1338 | 68 | 2685660 | 65 | 2156605 |
| 59 | 36349897 | 91.3235 | 64 | 2702850 | 69 | 2161781 |

| | | | | | | |
|---|---|---|---|---|---|---|
| 60 | 28949730 | 91.6194 | 67 | 2702850 | 71 | 2206436 |
| 61 | 36348782 | 93.1035 | 66 | 2702915 | 72 | 2206436 |
| 62 | 36346408 | 93.4151 | 65 | 2704980 | 73 | 2225964 |
| 63 | 36345705 | 94.0602 | 69 | 2710159 | 74 | 2247883 |
| 64 | 36350677 | 94.7452 | 71 | 2754842 | 76 | 2254200 |
| 65 | 36346198 | 95.0617 | 72 | 2754842 | 77 | 2256056 |
| 66 | 28946498 | 95.3822 | 73 | 2774435 | 78 | 2272302 |
| 67 | 28945928 | 96.0445 | 74 | 2796345 | 79 | 2276017 |
| 68 | 36346805 | 97.9441 | 76 | 2803366 | 83 | 2295438 |
| 69 | 28947843 | 100.5476 | 77 | 2805222 | 82 | 2297554 |
| 70 | 28949951 | 103.3898 | 78 | 2821942 | 80 | 2302444 |
| 71 | 28945295 | 103.7043 | 79 | 2825657 | 81 | 2305006 |
| 72 | 36347835 | 107.524 | 83 | 2845079 | 84 | 2331710 |
| 73 | 28946738 | 108.6938 | 80 | 2852062 | 85 | 2335131 |
| 74 | 36351186 | 109.2838 | 81 | 2854639 | 86 | 2341934 |
| 75 | 28947400 | 110.4502 | 84 | 2881396 | #N/A | |
| 76 | 28948460 | 110.7677 | 85 | 2885031 | #N/A | |
| 77 | 36345844 | 111.7387 | 86 | 2891845 | #N/A | |
| 78 | 28949379 | 112.0592 | #N/A | | #N/A | |

| | | | | | | |
|---|---|---|---|---|---|---|
| 79 | 36348990 | 113.749 | #N/A | | #N/A | |
| 80 | 28949631 | 114.378 | #N/A | | #N/A | |
| 81 | 36347533 | 114.9715 | #N/A | | #N/A | |
| 82 | 36348955 | 115.29 | #N/A | | #N/A | |
| 83 | 36346756 | 119.6053 | #N/A | | #N/A | |
| 84 | 36348608 | 120.57 | #N/A | | #N/A | |
| 85 | 28948910 | 121.1514 | #N/A | | #N/A | |
| 86 | 28949551 | 121.1515 | #N/A | | #N/A | |
| *Pop37_* Chr08 | | | W1-1 | | SG1 | |
| Marker order | Marker ID | cM | Marker order | Base pair | Marker order | Base pair |
| 1 | 36346627 | 0.637 | 4 | 474200 | 1 | 54382 |
| 2 | 28945978 | 0.9466 | 2 | 668580 | 5 | 182255 |
| 3 | 28946817 | 1.5383 | 1 | 778534 | 8 | 244875 |
| 4 | 36346579 | 2.1266 | 5 | 803753 | 7 | 259871 |
| 5 | 28950166 | 4.7529 | 8 | 887585 | 6 | 260781 |
| 6 | 36350523 | 5.0616 | 7 | 905417 | 9 | 274993 |
| 7 | 28948972 | 5.6885 | 6 | 910024 | 10 | 301182 |
| 8 | 28947643 | 6.2965 | 9 | 948668 | 11 | 359153 |

| | | | | | | |
|---|---|---|---|---|---|---|
| 9 | 28945819 | 7.4461 | 10 | 975086 | 15 | 658058 |
| 10 | 36350740 | 7.7402 | 11 | 1031764 | 14 | 730910 |
| 11 | 36347897 | 9.3433 | 13 | 1048360 | 13 | 749924 |
| 12 | 28949846 | 11.231 | 12 | 1265810 | 12 | 913590 |
| 13 | 28946729 | 11.5313 | 17 | 1284872 | 17 | 932683 |
| 14 | 36345758 | 11.8381 | 18 | 1312890 | 18 | 960698 |
| 15 | 36346057 | 13.9478 | 20 | 1348148 | 20 | 995955 |
| 16 | 36346479 | 14.8767 | 19 | 1376229 | 19 | 1023975 |
| 17 | 28945509 | 16.7124 | 21 | 1391665 | 21 | 1039411 |
| 18 | 28949106 | 20.7557 | 22 | 1397973 | 22 | 1045737 |
| 19 | 28949209 | 21.0682 | 23 | 1403381 | 23 | 1051145 |
| 20 | 36348042 | 22.3185 | 24 | 1494301 | 24 | 1133320 |
| 21 | 28947787 | 22.8949 | 25 | 1513615 | 25 | 1152621 |
| 22 | 28947058 | 23.7595 | 26 | 1563392 | 26 | 1202284 |
| 23 | 28946907 | 30.1901 | 27 | 1567984 | 27 | 1207189 |
| 24 | 28947674 | 31.6786 | 28 | 1586920 | 28 | 1226116 |
| 25 | 36347839 | 33.2082 | 29 | 1602031 | 29 | 1241226 |
| 26 | 36345660 | 33.5049 | 30 | 1604896 | 30 | 1244095 |
| 27 | 36351136 | 33.8089 | 31 | 1616206 | 31 | 1255428 |

| | | | | | | |
|---|---|---|---|---|---|---|
| 28 | 36350071 | 34.1137 | 32 | 1631873 | 32 | 1271093 |
| 29 | 28946265 | 35.0067 | 33 | 1667661 | 33 | 1312199 |
| 30 | 36345703 | 38.0133 | 34 | 1685112 | 34 | 1329643 |
| 31 | 36349684 | 39.2294 | 35 | 1703251 | 35 | 1347775 |
| 32 | 28947937 | 42.8186 | 37 | 1741918 | 37 | 1378118 |
| 33 | 28945657 | 45.8022 | 36 | 1743746 | 36 | 1379920 |
| 34 | 36345970 | 46.9965 | 38 | 1745431 | 38 | 1381605 |
| 35 | 36348680 | 48.4255 | 39 | 1776477 | 39 | 1412597 |
| 36 | 36350034 | 48.7162 | 40 | 1779226 | 40 | 1415342 |
| 37 | 28946530 | 49.022 | 41 | 1816580 | 41 | 1453923 |
| 38 | 36346930 | 50.5657 | 42 | 1827833 | 42 | 1465154 |
| 39 | 28947885 | 52.2846 | 43 | 1851297 | 43 | 1487752 |
| 40 | 36346154 | 53.288 | 45 | 1878861 | 45 | 1516573 |
| 41 | 36349770 | 55.4907 | 46 | 1897588 | 46 | 1535108 |
| 42 | 36345899 | 57.7724 | 47 | 1904662 | 47 | 1542164 |
| 43 | 36345894 | 58.7159 | 48 | 1911414 | 48 | 1548927 |
| 44 | 36346498 | 59.3008 | 49 | 1937875 | 49 | 1575416 |
| 45 | 28947818 | 59.8788 | 50 | 2111921 | 50 | 1689418 |
| 46 | 28948275 | 61.316 | 54 | 2111921 | 54 | 1689418 |

| | | | | | | |
|---|---|---|---|---|---|---|
| 47 | 36348689 | 61.6093 | 55 | 2997497 | 55 | 1889160 |
| 48 | 36348591 | 63.3799 | 53 | 3068419 | 53 | 1959878 |
| 49 | 28946642 | 64.5923 | 52 | 3080636 | 52 | 1972120 |
| 50 | 28947236 | 64.9019 | 57 | 3170375 | 57 | 2041430 |
| 51 | 36349042 | 65.1986 | 58 | 3179065 | 58 | 2049815 |
| 52 | 36346099 | 65.5161 | 59 | 3185886 | 59 | 2056636 |
| 53 | 36348589 | 66.1676 | 64 | 3204662 | 64 | 2075473 |
| 54 | 36345951 | 66.7985 | 60 | 3211603 | 60 | 2082587 |
| 55 | 28949194 | 67.3851 | 62 | 3214341 | 62 | 2085283 |
| 56 | 36350438 | 68.2701 | 63 | 3216214 | 65 | 2099413 |
| 57 | 28948406 | 68.8549 | 61 | 3217251 | 66 | 2128805 |
| 58 | 36347911 | 69.1517 | 65 | 3228485 | 67 | 2141715 |
| 59 | 28945556 | 69.7505 | 66 | 3257916 | 68 | 2155149 |
| 60 | 36350185 | 70.3855 | 67 | 3270847 | 69 | 2188069 |
| 61 | 36346949 | 71.0328 | 68 | 3284281 | 70 | 2188069 |
| 62 | 36349556 | 71.3472 | 69 | 3315525 | 73 | 2204154 |
| 63 | 36346927 | 71.6422 | 70 | 3315525 | 74 | 2209608 |
| 64 | 36346394 | 71.9321 | 73 | 3331661 | 77 | 2209608 |
| 65 | 28947140 | 72.2219 | 74 | 3337162 | 75 | 2264758 |

| | | | | | | |
|---|---|---|---|---|---|---|
| 66 | 36349453 | 72.5126 | 77 | 3337162 | 76 | 2282999 |
| 67 | 28948754 | 74.2729 | 75 | 3392311 | 78 | 2463181 |
| 68 | 28945966 | 76.0647 | 76 | 3410533 | 72 | 2479584 |
| 69 | 36347659 | 76.3606 | 78 | 3780665 | 71 | 2502539 |
| 70 | 36347658 | 78.1105 | 72 | 3797043 | 79 | 2543330 |
| 71 | 28947610 | 78.4127 | 71 | 3819947 | 81 | 2574601 |
| 72 | 36350697 | 79.0377 | 79 | 3860724 | 82 | 2575663 |
| 73 | 36346580 | 79.3542 | 82 | 3907344 | 80 | 2578595 |
| 74 | 36346706 | 79.6657 | 80 | 3910282 | 83 | 2693889 |
| 75 | 28945896 | 79.9589 | 85 | 3953116 | 84 | 2703475 |
| 76 | 28946856 | 80.2714 | 84 | 3962680 | 86 | 2723704 |
| 77 | 28946381 | 80.8907 | 86 | 3982857 | 87 | 2764005 |
| 78 | 28947618 | 81.4877 | 87 | 4023277 | 88 | 2777293 |
| 79 | 36348065 | 85.3761 | 88 | 4036560 | 89 | 2779985 |
| 80 | 36349715 | 85.6755 | 89 | 4039245 | 90 | 2786993 |
| 81 | 36350904 | 86.0755 | 90 | 4046257 | 91 | 2818792 |
| 82 | 28946207 | 88.5265 | 91 | 4077797 | 93 | 2889353 |
| 83 | 36348927 | 89.14 | 92 | 4114650 | 94 | 2892572 |
| 84 | 36353420 | 89.4421 | 93 | 4152060 | 95 | 2925360 |

| | | | | | | |
|---|---|---|---|---|---|---|
| 85 | 28946348 | 89.7452 | 94 | 4155277 | 97 | 2973928 |
| 86 | 28945743 | 90.9466 | 95 | 4188014 | 96 | 2976572 |
| 87 | 28945903 | 91.2469 | 97 | 4230004 | 98 | 2985137 |
| 88 | 28946732 | 91.5351 | 96 | 4232648 | 101 | 2985137 |
| 89 | 28947672 | 92.2842 | 98 | 4241148 | 99 | 2985352 |
| 90 | 41804206 | 94.9382 | 101 | 4241148 | 103 | 3005327 |
| 91 | 28946492 | 97.417 | 99 | 4241363 | 104 | 3034700 |
| 92 | 28950231 | 98.6443 | 103 | 4261339 | 102 | 3060013 |
| 93 | 28946434 | 99.2752 | 104 | 4291670 | 105 | 3077698 |
| 94 | 36347660 | 101.801 | 102 | 4316786 | 106 | 3092215 |
| 95 | 36349437 | 102.0876 | 106 | 4349967 | 107 | 3130281 |
| 96 | 36347597 | 102.3962 | 107 | 4388051 | 108 | 3179690 |
| 97 | 36346664 | 102.7219 | 108 | 4437614 | 109 | 3182117 |
| 98 | 36349686 | 103.0404 | 109 | 4440045 | 110 | 3206937 |
| 99 | 28946840 | 103.3311 | 110 | 4464918 | 112 | 3243925 |
| 100 | 36351016 | 103.9409 | 112 | 4501979 | 113 | 3246774 |
| 101 | 28946557 | 105.4942 | 113 | 4504817 | 114 | 3287906 |
| 102 | 28945554 | 106.729 | 114 | 4545917 | 115 | 3294723 |
| 103 | 36347661 | 107.0206 | 115 | 4552938 | 116 | 3300840 |

| | | | | | | |
|---|---|---|---|---|---|---|
| 104 | 28945288 | 107.3138 | 116 | 4559078 | 117 | 3315875 |
| 105 | 36348890 | 109.3618 | 117 | 4574134 | 118 | 3348740 |
| 106 | 28948907 | 110.2264 | 118 | 4607456 | 119 | 3389258 |
| 107 | 28948082 | 111.2037 | 119 | 4647983 | 120 | 3399030 |
| 108 | 36345662 | 111.5284 | 120 | 4657753 | 121 | 3403537 |
| 109 | 28946288 | 112.6847 | 121 | 4662258 | 122 | 3474270 |
| 110 | 36349566 | 113.2747 | 122 | 4685287 | 123 | 3475593 |
| 111 | 36346712 | 113.8612 | 123 | 4686610 | 124 | 3579553 |
| 112 | 28948231 | 115.2984 | 124 | 4790660 | 125 | 3584617 |
| 113 | 36348202 | 116.4547 | 125 | 4795705 | 126 | 3644762 |
| 114 | 28945422 | 116.7454 | 126 | 4855795 | 127 | 3669736 |
| 115 | 28947704 | 117.9016 | 127 | 4880727 | 128 | 3698159 |
| 116 | 28946919 | 118.5427 | 128 | 4909159 | 129 | 3763750 |
| 117 | 36346386 | 119.1838 | 129 | 4974708 | 132 | 3809818 |
| 118 | 36347694 | 121.7873 | 132 | 5020732 | 130 | 3820176 |
| 119 | 36348044 | 122.0797 | 130 | 5031094 | 134 | 3933380 |
| 120 | 36348049 | 122.7047 | 133 | 5121704 | 135 | 3970227 |
| 121 | 36349452 | 123.3458 | 134 | 5137703 | 136 | 3984579 |
| 122 | 36348897 | 123.6573 | 135 | 5174522 | 138 | 4056673 |

| | | | | | | |
|---|---|---|---|---|---|---|
| 123 | 28947972 | 124.5476 | 136 | 5190513 | 139 | 4080568 |
| 124 | 28947539 | 125.1256 | 137 | 5223069 | 141 | 4080568 |
| 125 | 28946205 | 125.4305 | 138 | 5262592 | 140 | 4133804 |
| 126 | 36347607 | 125.7373 | #N/A | 5276503 | 147 | 4195622 |
| 127 | 36350543 | 126.8969 | 139 | 5309158 | 148 | 4243562 |
| 128 | 28947590 | 127.4733 | 141 | 5309158 | 145 | 4251168 |
| 129 | 36348234 | 129.7938 | 140 | 5353602 | 144 | 4263440 |
| 130 | 36347718 | 130.0837 | 147 | 5529872 | 149 | 4269221 |
| 131 | 28946792 | 132.2794 | 148 | 5577943 | 143 | 4284027 |
| 132 | 28945363 | 134.2348 | 145 | 5585546 | 146 | 4316628 |
| 133 | 36349596 | 136.0933 | 144 | 5600584 | 150 | 4331596 |
| 134 | 36348334 | 136.3814 | 143 | 5620971 | 152 | 4352777 |
| 135 | 28946861 | 137.2486 | 142 | 5645050 | 151 | 4358380 |
| 136 | 36349046 | 138.9784 | 146 | 5653572 | 153 | 4385751 |
| 137 | 28949891 | 143.0119 | 150 | 5668550 | 154 | 4420785 |
| 138 | 36345872 | 143.3355 | 152 | 5689810 | 155 | 4545015 |
| 139 | 36347964 | 144.9233 | 151 | 5698704 | 157 | 4647899 |
| 140 | 28946175 | 148.3236 | 153 | 5725945 | 156 | 4658799 |
| 141 | 36347965 | 153.2253 | 154 | 5761207 | 160 | 4711299 |

| | | | | | | |
|---|---|---|---|---|---|---|
| 142 | 36350274 | 153.5586 | 155 | 5792850 | 161 | 4768710 |
| 143 | 28945708 | 156.1249 | 157 | 5936127 | 162 | 4788442 |
| 144 | 28949835 | 158.3276 | 156 | 5947033 | 163 | 4811243 |
| 145 | 36347585 | 160.5029 | 160 | 5999571 | 165 | 4905817 |
| 146 | 28946704 | 161.3651 | 161 | 6007171 | 167 | 4916765 |
| 147 | 28947925 | 161.9881 | 162 | 6026730 | 164 | 4919725 |
| 148 | 36348014 | 162.3006 | 163 | 6049592 | 166 | 4919725 |
| 149 | 36348415 | 163.2531 | 165 | 6175967 | 170 | 4941696 |
| 150 | 36350611 | 166.5803 | 167 | 6186842 | 172 | 4959218 |
| 151 | 36346938 | 166.9125 | 164 | 6189805 | 171 | 4976837 |
| 152 | 36346460 | 167.5435 | 166 | 6189805 | 174 | 5082263 |
| 153 | 36351265 | 169.864 | 170 | 6211777 | 176 | 5082267 |
| 154 | 36348740 | 170.7438 | 172 | 6229303 | 173 | 5089755 |
| 155 | 28946560 | 173.2538 | 175 | 6333503 | 177 | 5099367 |
| 156 | 28945919 | 173.8828 | 174 | 6349330 | 178 | 5112821 |
| 157 | 36348627 | 175.4312 | 176 | 6349334 | 181 | 5136504 |
| 158 | 36350386 | 176.363 | 173 | 6356824 | 180 | 5142055 |
| 159 | 36349244 | 177.5328 | 177 | 6366404 | 184 | 5161262 |
| 160 | 28950119 | 178.1176 | 178 | 6379857 | 183 | 5166196 |

| | | | | | | |
|---|---|---|---|---|---|---|
| 161 | 36350090 | 179.5974 | 179 | 6388412 | 182 | 5170996 |
| 162 | 36349857 | 181.4443 | 181 | 6403501 | 185 | 5216727 |
| 163 | 28948445 | 186.6142 | 180 | 6409053 | 186 | 5257520 |
| 164 | 36348791 | 186.9032 | 184 | 6428226 | 187 | 5262112 |
| 165 | 28948106 | 187.2237 | 183 | 6433153 | 188 | 5273404 |
| 166 | 36348790 | 187.556 | 182 | 6437931 | 189 | 5283735 |
| 167 | 36346034 | 189.4556 | 185 | 6483545 | 190 | 5285988 |
| 168 | 36350428 | 190.0599 | 186 | 6523529 | 191 | 5303874 |
| 169 | 36349239 | 190.9321 | 187 | 6528292 | 192 | 5330958 |
| 170 | 28948346 | 191.5571 | 188 | 6539533 | 193 | 5335567 |
| 171 | 36348993 | 191.8766 | 189 | 6549854 | #N/A | |
| 172 | 36346785 | 193.5722 | 190 | 6552101 | #N/A | |
| 173 | 36348226 | 194.5893 | 191 | 6569974 | #N/A | |
| 174 | 36345945 | 195.4743 | 192 | 6596227 | #N/A | |
| 175 | 36348767 | 196.0804 | #N/A | | #N/A | |
| 176 | 36346758 | 197.6146 | #N/A | | #N/A | |
| 177 | 36349852 | 198.4971 | #N/A | | #N/A | |
| 178 | 28948652 | 199.3718 | #N/A | | #N/A | |
| 179 | 36350545 | 202.6021 | #N/A | | #N/A | |

| | | | | | | |
|---|---|---|---|---|---|---|
| 180 | 28947300 | 202.9107 | #N/A | | #N/A | |
| 181 | 28946215 | 204.8661 | #N/A | | #N/A | |
| 182 | 36349531 | 206.784 | #N/A | | #N/A | |
| 183 | 28949012 | 207.0907 | #N/A | | #N/A | |
| 184 | 36350783 | 216.8398 | #N/A | | #N/A | |
| 185 | 36347716 | 219.4893 | #N/A | | #N/A | |
| 186 | 36348347 | 220.0707 | #N/A | | #N/A | |
| 187 | 28948112 | 220.659 | #N/A | | #N/A | |
| 188 | 28947481 | 221.5415 | #N/A | | #N/A | |
| 189 | 36348325 | 222.7011 | #N/A | | #N/A | |
| 190 | 28946815 | 223.0165 | #N/A | | #N/A | |
| 191 | 36348963 | 229.7447 | #N/A | | #N/A | |
| 192 | 36350229 | 230.0612 | #N/A | | #N/A | |
| 193 | 36346769 | 230.0613 | #N/A | | #N/A | |
| *Pop37*_ Chr09 | | | W1-1 | | SG1 | |
| Marker order | Marker ID | cM | Marker order | Base pair | Marker order | Base pair |
| 1 | 36348957 | 0.6452 | 7 | 476594 | 7 | 75371 |
| 2 | 28949238 | 1.8015 | 6 | 492038 | 5 | 132254 |

| | | | | | | |
|---|---|---|---|---|---|---|
| 3 | 28946611 | 2.388 | 5 | 533347 | 4 | 146991 |
| 4 | 28947473 | 2.9745 | 4 | 548061 | 3 | 161078 |
| 5 | 36350573 | 4.5135 | 3 | 561231 | 9 | 188180 |
| 6 | 28949660 | 4.8632 | 9 | 588000 | 10 | 202990 |
| 7 | 28947207 | 5.208 | 10 | 614084 | 11 | 245928 |
| 8 | 36349284 | 8.1613 | 11 | 628463 | 2 | 246206 |
| 9 | 28947554 | 8.7444 | 2 | 628741 | 1 | 248949 |
| 10 | 28948150 | 9.3896 | 1 | 631483 | 14 | 330985 |
| 11 | 28949657 | 9.7143 | 12 | 645242 | 13 | 356760 |
| 12 | 36350663 | 10.3095 | 14 | 685053 | 21 | 467875 |
| 13 | 28949093 | 11.552 | 13 | 710685 | 15 | 473133 |
| 14 | 36347982 | 17.0402 | 21 | 807352 | 20 | 476518 |
| 15 | 36345667 | 17.3577 | 15 | 812576 | 19 | 497735 |
| 16 | 36346286 | 17.6771 | 20 | 815968 | 18 | 502895 |
| 17 | 36349712 | 17.9766 | 19 | 837176 | 17 | 505180 |
| 18 | 28946882 | 18.2623 | 18 | 842329 | 22 | 507712 |
| 19 | 36348559 | 18.5505 | 17 | 844614 | 16 | 519093 |
| 20 | 28945838 | 19.4408 | 22 | 847146 | 23 | 548578 |
| 21 | 28946276 | 20.6174 | 16 | 858531 | 24 | 699097 |

| | | | | | | |
|---|---|---|---|---|---|---|
| 22 | 28946157 | 21.561 | 23 | 887995 | 25 | 702117 |
| 23 | 36347985 | 22.532 | 24 | 952297 | 27 | 734055 |
| 24 | 36350769 | 23.4844 | 25 | 955322 | 28 | 734586 |
| 25 | 36346635 | 28.8851 | 27 | 987231 | 26 | 769405 |
| 26 | 36346755 | 29.5141 | 28 | 987772 | 29 | 780598 |
| 27 | 36348803 | 29.8126 | 26 | 1021939 | 30 | 802843 |
| 28 | 36350779 | 30.6698 | 29 | 1033141 | 31 | 841710 |
| 29 | 28946661 | 31.8396 | 30 | 1055386 | 32 | 876223 |
| 30 | 36350521 | 35.0604 | 31 | 1094283 | 33 | 901217 |
| 31 | 28947147 | 36.5402 | 32 | 1171258 | 34 | 916568 |
| 32 | 36348095 | 37.139 | 33 | 1196189 | 36 | 956496 |
| 33 | 36349927 | 42.1057 | 34 | 1211494 | 37 | 956496 |
| 34 | 28945218 | 47.2829 | 36 | 1251226 | 35 | 978963 |
| 35 | 36346542 | 47.6326 | 37 | 1251226 | 38 | 1001753 |
| 36 | 28948536 | 47.9311 | 35 | 1273721 | 41 | 1005083 |
| 37 | 36352131 | 48.8716 | 38 | 1297272 | 43 | 1020229 |
| 38 | 36345766 | 49.487 | 41 | 1300607 | 39 | 1020599 |
| 39 | 28948243 | 50.3492 | 43 | 1315764 | 42 | 1029905 |
| 40 | 100311996 | 51.2342 | 39 | 1316134 | 40 | 1055319 |

| | | | | | | |
|---|---|---|---|---|---|---|
| 41 | 28945996 | 51.5953 | 42 | 1325452 | 45 | 1066889 |
| 42 | 36346129 | 51.9769 | 40 | 1351129 | 47 | 1077937 |
| 43 | 28945563 | 52.9906 | 45 | 1362697 | 46 | 1083729 |
| 44 | 36346772 | 54.3608 | 47 | 1373719 | 49 | 1110041 |
| 45 | 36350800 | 57.194 | 46 | 1379511 | 48 | 1126703 |
| 46 | 36349435 | 57.7771 | 49 | 1405838 | 52 | 1188238 |
| 47 | 28948667 | 59.2226 | 52 | 1443820 | 50 | 1212268 |
| 48 | 36350717 | 60.1374 | 50 | 1467848 | 51 | 1221718 |
| 49 | 41804965 | 61.9674 | 51 | 1477298 | 53 | 1244966 |
| 50 | 36348476 | 62.2548 | 53 | 1500532 | 54 | 1264166 |
| 51 | 28947727 | 63.8081 | 54 | 1519737 | 55 | 1282682 |
| 52 | 36346119 | 65.0782 | 55 | 1538259 | 56 | 1327233 |
| 53 | 28950088 | 66.5938 | 56 | 1584653 | 62 | 1337030 |
| 54 | 36347887 | 68.375 | 62 | 1594426 | 57 | 1337033 |
| 55 | 36347691 | 73.3476 | 57 | 1594429 | 63 | 1354675 |
| 56 | 28948223 | 73.95 | 63 | 1612081 | 58 | 1354676 |
| 57 | 28947498 | 74.2737 | 58 | 1612082 | 61 | 1367397 |
| 58 | 36345840 | 75.2606 | 61 | 1624799 | 60 | 1379708 |
| 59 | 36346098 | 76.8434 | 60 | 1637151 | 59 | 1398506 |

| | | | | | | |
|---|---|---|---|---|---|---|
| 60 | 28947636 | 77.1511 | 59 | 1655945 | 68 | 1430456 |
| 61 | 28946702 | 78.0659 | 66 | 1679498 | 66 | 1450708 |
| 62 | 28945447 | 78.7195 | 65 | 2184617 | 65 | 1803453 |
| 63 | 36346557 | 81.7231 | 67 | 2238857 | 67 | 1856543 |
| 64 | 36346629 | 82.0531 | 64 | 2270283 | 64 | 1888050 |
| 65 | 28945908 | 83.0179 | 70 | 2270348 | 70 | 1888112 |
| 66 | 28946982 | 83.3061 | 71 | 2309120 | 71 | 1906757 |
| 67 | 28947961 | 83.6028 | 72 | 2342946 | 72 | 1937866 |
| 68 | 36346011 | 83.9031 | 74 | 2603650 | 74 | 2005272 |
| 69 | 28949077 | 84.1955 | 76 | 2616297 | 76 | 2017918 |
| 70 | 28949346 | 85.0702 | 75 | 2643211 | 75 | 2043505 |
| 71 | 36349736 | 87.1063 | 78 | 3008642 | 79 | 2082777 |
| 72 | 28947115 | 88.5948 | #N/A | | 78 | 2093550 |
| 73 | 28946113 | 90.1385 | #N/A | | #N/A | |
| 74 | 36347728 | 91.7314 | #N/A | | #N/A | |
| 75 | 36349716 | 92.3684 | #N/A | | #N/A | |
| 76 | 28948413 | 92.6582 | #N/A | | #N/A | |
| 77 | 36351047 | 94.6825 | #N/A | | #N/A | |
| 78 | 28947470 | 95.5916 | #N/A | | #N/A | |

| | | | | | | |
|---|---|---|---|---|---|---|
| 79 | 36346461 | 95.5917 | #N/A | | #N/A | |
| *Pop37_* Chr10 | | | W1-1 | | SG1 | |
| Marker order | Marker ID | cM | Marker order | Base pair | Marker order | Base pair |
| 1 | 36346903 | 0.6645 | 2 | 58194 | 2 | 144146 |
| 2 | 36346882 | 1.608 | 4 | 88073 | 4 | 185718 |
| 3 | 28947075 | 2.1861 | 5 | 93453 | 5 | 191098 |
| 4 | 28947397 | 2.5056 | 6 | 93851 | 6 | 191496 |
| 5 | 36346674 | 2.8705 | 1 | 97694 | 1 | 195309 |
| 6 | 36346752 | 4.7441 | 7 | 97694 | 7 | 195309 |
| 7 | 36346404 | 7.0258 | 8 | 103577 | 9 | 201196 |
| 8 | 28948447 | 7.3442 | 10 | 150815 | 10 | 248983 |
| 9 | 36350675 | 20.4918 | 11 | 189893 | 11 | 287613 |
| 10 | 28949410 | 24.1457 | 12 | 193527 | 12 | 291249 |
| 11 | 36346082 | 24.4469 | 13 | 195379 | 13 | 293101 |
| 12 | 28947224 | 25.0386 | 16 | 216083 | 15 | 301065 |
| 13 | 28948270 | 28.0007 | 17 | 216554 | 14 | 301130 |
| 14 | 36348965 | 28.2992 | 18 | 223611 | 16 | 313937 |
| 15 | 36346754 | 29.1975 | 19 | 224088 | 17 | 314409 |

| | | | | | | |
|---|---|---|---|---|---|---|
| 16 | 28948094 | 29.489 | 20 | 233394 | 18 | 321474 |
| 17 | 36349500 | 32.3908 | 21 | 278381 | 19 | 321951 |
| 18 | 28945894 | 32.6815 | 22 | 322497 | 20 | 331290 |
| 19 | 36349465 | 34.7355 | 23 | 343860 | 21 | 375422 |
| 20 | 28948384 | 41.5016 | 24 | 487028 | 22 | 419610 |
| 21 | 28948435 | 43.5259 | 25 | 513101 | 25 | 478075 |
| 22 | 28946487 | 46.1676 | 26 | 576578 | 26 | 537812 |
| 23 | 28948546 | 47.0553 | 30 | 610936 | 31 | 539882 |
| 24 | 36345936 | 49.6893 | 29 | 616109 | 30 | 571987 |
| 25 | 28946659 | 50.604 | 27 | 727908 | 29 | 577101 |
| 26 | 28945841 | 51.2775 | 34 | 863213 | 28 | 587688 |
| 27 | 36346524 | 51.6119 | 35 | 893280 | 27 | 927208 |
| 28 | 36346560 | 51.9304 | 33 | 932363 | 34 | 946364 |
| 29 | 36346660 | 53.1652 | 36 | 936605 | 35 | 966640 |
| 30 | 28948257 | 55.0237 | 37 | 962811 | 33 | 1005848 |
| 31 | 36345992 | 56.3354 | 32 | 965450 | 36 | 1010075 |
| 32 | 41804214 | 57.9386 | 38 | 973679 | 37 | 1036270 |
| 33 | 28947265 | 58.2267 | 39 | 985425 | 32 | 1038905 |
| 34 | 28947687 | 58.5166 | 40 | 1024844 | 38 | 1047134 |

| | | | | | | |
|---|---|---|---|---|---|---|
| 35 | 28948154 | 58.8206 | 44 | 1024844 | 39 | 1058828 |
| 36 | 36348760 | 59.7297 | 43 | 1029421 | 40 | 1098043 |
| 37 | 28947603 | 60.03 | 41 | 1080743 | 44 | 1098043 |
| 38 | 36346506 | 60.9338 | 45 | 1082707 | 43 | 1102621 |
| 39 | 28947006 | 62.4223 | 49 | 1141250 | 41 | 1153568 |
| 40 | 28946220 | 63.3315 | 46 | 1197438 | 45 | 1155527 |
| 41 | 28945257 | 63.6318 | 48 | 1208744 | 49 | 1438794 |
| 42 | 28949616 | 63.9242 | 51 | 1210399 | 46 | 1762763 |
| 43 | 28947851 | 66.6877 | 50 | 1211809 | 48 | 1774083 |
| 44 | 36348005 | 67.0156 | 47 | 1236125 | 51 | 1775738 |
| 45 | 36345888 | 67.6846 | 52 | 1236125 | 50 | 1777151 |
| 46 | 36346367 | 68.3256 | 53 | 1241815 | 47 | 1801497 |
| 47 | 36350070 | 69.8231 | 54 | 1256159 | 52 | 1801497 |
| 48 | 28947189 | 70.4113 | 55 | 1285941 | 53 | 1807203 |
| 49 | 36348078 | 71.7019 | 56 | 1295097 | 54 | 1821427 |
| 50 | 28946331 | 72.0235 | 57 | 1386733 | 55 | 1851182 |
| 51 | 28946040 | 72.6564 | 58 | 1453355 | 56 | 1860412 |
| 52 | 28945724 | 72.9719 | 59 | 1508413 | 57 | 1913423 |
| 53 | 28946051 | 73.2731 | 61 | 1691272 | 58 | 2008283 |

| | | | | | | |
|---|---|---|---|---|---|---|
| 54 | 36350127 | 74.1795 | 62 | 1713224 | 59 | 2063095 |
| 55 | 28949383 | 74.4745 | 64 | 1719522 | 61 | 2239921 |
| 56 | 28947339 | 75.6209 | 63 | 1924661 | 62 | 2261814 |
| 57 | 28946683 | 77.3358 | 65 | 1952836 | 64 | 2268112 |
| 58 | 28946131 | 79.0858 | 66 | 1970888 | 63 | 2571955 |
| 59 | 28949452 | 80.2695 | 67 | 1994211 | 65 | 2600099 |
| 60 | 28949317 | 81.7943 | 68 | 2003917 | 66 | 2618205 |
| 61 | 28947389 | 82.0955 | 69 | 2023119 | 67 | 2641541 |
| 62 | 28947128 | 82.3977 | 70 | 2030321 | 68 | 2651243 |
| 63 | 36351591 | 83.0001 | 71 | 2043344 | 69 | 2670129 |
| 64 | 28947894 | 83.5782 | 72 | 2043344 | 70 | 2677393 |
| 65 | 28947630 | 84.4428 | 73 | 2061493 | 71 | 2690571 |
| 66 | 36349067 | 85.6195 | 74 | 2069129 | 72 | 2690571 |
| 67 | 36348013 | 86.2256 | 75 | 2150276 | 73 | 2708659 |
| 68 | 36347832 | 87.7693 | #N/A | | 75 | 2720455 |
| 69 | 36349797 | 91.8407 | #N/A | | #N/A | |
| 70 | 36348729 | 93.705 | #N/A | | #N/A | |
| 71 | 36346967 | 94.3339 | #N/A | | #N/A | |
| 72 | 36347849 | 95.5574 | #N/A | | #N/A | |

| | | | | | | |
|---|---|---|---|---|---|---|
| 73 | 28948651 | 96.1474 | #N/A | | #N/A | |
| 74 | 28945403 | 97.3206 | #N/A | | #N/A | |
| 75 | 36350050 | 97.3207 | #N/A | | #N/A | |
| *Pop37_* Chr11 | | | W1-1 | | SG1 | |
| Marker order | Marker ID | cM | Marker order | Base pair | Marker order | Base pair |
| 1 | 28945322 | 0.3135 | 1 | 109004 | 4 | 58446 |
| 2 | 36346728 | 4.8995 | 4 | 111740 | 5 | 60306 |
| 3 | 36346663 | 5.5056 | 5 | 113600 | 1 | 65129 |
| 4 | 28945717 | 5.8152 | #N/A | 149048 | 6 | 250568 |
| 5 | 28945140 | 6.1146 | 7 | 200745 | 7 | 277988 |
| 6 | 36351344 | 8.1746 | 8 | 235921 | 8 | 313236 |
| 7 | 28948432 | 10.4817 | 9 | 241491 | 9 | 318802 |
| 8 | 36349809 | 11.6904 | 10 | 252339 | 10 | 329457 |
| 9 | 36347703 | 12.3174 | 13 | 256488 | 13 | 333596 |
| 10 | 36346565 | 13.2321 | 14 | 264779 | 14 | 341889 |
| 11 | 36346575 | 13.5219 | 12 | 277816 | 12 | 354911 |
| 12 | 28947032 | 14.7198 | 19 | 363304 | 11 | 358480 |
| 13 | 36351335 | 15.0466 | 17 | 487067 | 15 | 394230 |

| | | | | | | |
|---|---|---|---|---|---|---|
| 14 | 36348458 | 18.3408 | 15 | 593148 | 20 | 432173 |
| 15 | 28945461 | 18.6573 | 16 | 930042 | 21 | 432173 |
| 16 | 36345831 | 19.5637 | 20 | 955736 | 22 | 500250 |
| 17 | 36352440 | 19.8649 | 21 | 955736 | 24 | 514245 |
| 18 | 36352928 | 20.1643 | 22 | 1043466 | 23 | 516338 |
| 19 | 36351841 | 21.3992 | 24 | 1057421 | 25 | 524965 |
| 20 | 36350556 | 21.704 | 23 | 1059514 | 26 | 538775 |
| 21 | 28947807 | 24.2851 | 25 | 1068112 | 28 | 563634 |
| 22 | 28946735 | 24.8876 | 26 | 1081905 | 27 | 585461 |
| 23 | 36346059 | 25.1924 | 28 | 1106805 | 29 | 604482 |
| 24 | 36348250 | 28.137 | 27 | 1128633 | 30 | 660163 |
| 25 | 36348297 | 30.5444 | 29 | 1147649 | 31 | 677385 |
| 26 | 36347800 | 32.6962 | 30 | 1203353 | 32 | 700846 |
| 27 | 28947593 | 33.0068 | 31 | 1220591 | 33 | 790932 |
| 28 | 28946555 | 33.8832 | 32 | 1244034 | 34 | 877376 |
| 29 | 28945715 | 36.4943 | 33 | 1363227 | 35 | 902737 |
| 30 | 36350583 | 36.7992 | 34 | 1393963 | 36 | 934854 |
| 31 | 36349781 | 39.0515 | 35 | 1419351 | 37 | 974406 |
| 32 | 36348645 | 41.22 | 36 | 1451414 | 38 | 995873 |

| | | | | | | |
|---|---|---|---|---|---|---|
| 33 | 36349639 | 43.0012 | 37 | 1593053 | 39 | 1060315 |
| 34 | 36348853 | 48.1642 | 38 | 1614534 | 41 | 1111240 |
| 35 | 36345715 | 51.9911 | 39 | 1679092 | 42 | 1124714 |
| 36 | 36346796 | 54.2434 | 41 | 1730005 | 43 | 1141001 |
| 37 | 36347680 | 54.5384 | 42 | 1743461 | 44 | 1143578 |
| 38 | 28947305 | 55.1115 | 43 | 1759754 | 45 | 1185000 |
| 39 | 36350741 | 55.698 | 44 | 1762334 | 47 | 1262481 |
| 40 | 36349275 | 57.7888 | 45 | 1801400 | 49 | 1262481 |
| 41 | 36352496 | 61.8223 | 47 | 1870507 | 48 | 1301658 |
| 42 | 36345723 | 62.1428 | 49 | 1870507 | 50 | 1322309 |
| 43 | 36345913 | 62.4623 | 48 | 1917523 | 51 | 1387549 |
| 44 | 36350400 | 66.0398 | 50 | 1941303 | 52 | 1440997 |
| 45 | 36351194 | 69.0555 | 51 | 2099740 | 53 | 1517245 |
| 46 | 36346815 | 70.3673 | 52 | 2153190 | 54 | 1536430 |
| 47 | 36350787 | 72.9254 | 53 | 2527472 | 56 | 1541420 |
| 48 | 36347662 | 75.3739 | 54 | 2546677 | 55 | 1572820 |
| 49 | 36350786 | 77.8681 | 56 | 2551673 | 57 | 1594074 |
| 50 | 28947865 | 79.4868 | 55 | 2583074 | 58 | 1607028 |
| 51 | 36347610 | 80.1558 | 57 | 2604083 | 59 | 1632338 |

| | | | | | | |
|---|---|---|---|---|---|---|
| 52 | 36348989 | 81.0818 | 58 | 2617003 | 60 | 1649243 |
| 53 | 28946130 | 81.7068 | 59 | 2642483 | 61 | 1684761 |
| 54 | 36351306 | 84.549 | 60 | 2659323 | 63 | 1700819 |
| 55 | 28949788 | 86.1072 | 61 | 2694750 | 67 | 1714788 |
| 56 | 36346795 | 88.7586 | 62 | 2710864 | 65 | 1728566 |
| 57 | 36346762 | 90.1242 | 67 | 2724828 | 64 | 1735445 |
| 58 | 36349427 | 91.419 | 65 | 2738609 | 66 | 1787710 |
| 59 | 36350233 | 92.3365 | 64 | 2745498 | 68 | 1795774 |
| 60 | 28949088 | 94.9782 | 66 | 2797750 | 80 | 1826003 |
| 61 | 28950234 | 95.2804 | 68 | 2805583 | 81 | 1831952 |
| 62 | 36347696 | 95.5968 | 80 | 2836029 | 69 | 1841730 |
| 63 | 36346540 | 96.8916 | 81 | 2841983 | 74 | 1886133 |
| 64 | 36346608 | 97.2195 | 69 | 2912239 | 79 | 1903835 |
| 65 | 36346675 | 97.5452 | 74 | 2956651 | 70 | 1911472 |
| 66 | 36348816 | 98.4977 | 79 | 2973982 | 71 | 1917962 |
| 67 | 28947834 | 101.3827 | 70 | 2981643 | 72 | 2225238 |
| 68 | 36349498 | 104.1463 | 71 | 2988118 | 75 | 2227605 |
| 69 | 36346586 | 104.7694 | 73 | 3451446 | #N/A | |
| 70 | 36351571 | 105.1678 | 78 | 3483218 | #N/A | |

| | | | | | | |
|---|---|---|---|---|---|---|
| 71 | 28947175 | 106.3777 | 72 | 3567170 | #N/A | |
| 72 | 36346742 | 107.0271 | 75 | 3569620 | #N/A | |
| 73 | 36350118 | 107.3161 | 76 | 3772727 | #N/A | |
| 74 | 28948778 | 107.8942 | 77 | 3772792 | #N/A | |
| 75 | 36350073 | 109.3523 | #N/A | | #N/A | |
| 76 | 36348520 | 109.9813 | #N/A | | #N/A | |
| 77 | 36347586 | 111.6 | #N/A | | #N/A | |
| 78 | 36345739 | 112.5585 | #N/A | | #N/A | |
| 79 | 36346360 | 113.1955 | #N/A | | #N/A | |
| 80 | 28946573 | 113.5032 | #N/A | | #N/A | |
| 81 | 36346512 | 113.5033 | #N/A | | #N/A | |
| *Pop37_* Chr12 | | | W1-1 | | SG1 | |
| Marker order | Marker ID | cM | Marker order | Base pair | Marker order | Base pair |
| 1 | 36352523 | 1.1301 | 5 | 361269 | 3 | 63893 |
| 2 | 36348996 | 1.4269 | 6 | 367388 | 5 | 128854 |
| 3 | 36346906 | 1.7236 | 7 | 418227 | 6 | 134954 |
| 4 | 36349007 | 2.6301 | 8 | 442373 | 7 | 175165 |
| 5 | 36346885 | 3.2307 | 9 | 508637 | 8 | 199339 |

| | | | | | | |
|---|---|---|---|---|---|---|
| 6 | 28950051 | 5.5512 | 10 | 529047 | 9 | 213609 |
| 7 | 28945529 | 9.3125 | 11 | 566373 | 10 | 234032 |
| 8 | 36347701 | 9.6586 | 12 | 596200 | 11 | 271327 |
| 9 | 36348695 | 12.3885 | 13 | 612740 | 12 | 293547 |
| 10 | 28948204 | 18.4237 | 14 | 623738 | 13 | 310095 |
| 11 | 36352145 | 19.0298 | 15 | 639344 | 14 | 321080 |
| 12 | 36350526 | 19.9201 | 17 | 648662 | 15 | 336682 |
| 13 | 28947913 | 20.5083 | 19 | 661168 | 17 | 346000 |
| 14 | 36348515 | 21.4093 | 18 | 669249 | 16 | 347190 |
| 15 | 36347711 | 21.7199 | 21 | 684446 | 19 | 358577 |
| 16 | 36347688 | 22.0276 | 22 | 692386 | 18 | 366664 |
| 17 | 28948663 | 22.609 | 26 | 694967 | 21 | 381868 |
| 18 | 36350785 | 23.5322 | 23 | 730801 | 22 | 389733 |
| 19 | 36348898 | 24.4817 | 27 | 730806 | 26 | 392312 |
| 20 | 36346646 | 26.4057 | 28 | 746404 | 23 | 428171 |
| 21 | 36347902 | 27.7089 | 25 | 768266 | 27 | 428176 |
| 22 | 36345910 | 28.6768 | 24 | 775512 | 28 | 443760 |
| 23 | 36348429 | 29.298 | 29 | 1136825 | 25 | 465587 |
| 24 | 36345775 | 30.2071 | 30 | 1216250 | 24 | 472782 |

| | | | | | | |
|---|---|---|---|---|---|---|
| 25 | 28945829 | 30.7954 | 31 | 1266408 | 29 | 806777 |
| 26 | 28948924 | 31.119 | 32 | 1280178 | 30 | 875014 |
| 27 | 28950055 | 31.7642 | 33 | 1333853 | 31 | 924900 |
| 28 | 36348814 | 32.07 | 34 | 1346274 | 32 | 938687 |
| 29 | 36350077 | 33.7998 | 35 | 1374977 | 33 | 964078 |
| 30 | 28946466 | 34.9462 | 36 | 1415143 | 34 | 976406 |
| 31 | 28946127 | 35.2335 | 37 | 1422520 | 35 | 1005108 |
| 32 | 36348568 | 38.3528 | 38 | 1430276 | 36 | 1045260 |
| 33 | 36345654 | 40.2893 | 39 | 1463568 | 37 | 1052637 |
| 34 | 28945368 | 41.5053 | 40 | 1478980 | 38 | 1060396 |
| 35 | 36348368 | 42.1114 | 41 | 1518212 | 39 | 1093708 |
| 36 | 28945544 | 43.9086 | 42 | 1530623 | 40 | 1109128 |
| 37 | 28945284 | 44.201 | 43 | 1568200 | 41 | 1150374 |
| 38 | 28946307 | 45.0656 | 44 | 1597109 | 42 | 1162752 |
| 39 | 28945975 | 45.6404 | 45 | 1665394 | 43 | 1200238 |
| 40 | 28946998 | 46.5025 | 46 | 1669454 | 44 | 1229340 |
| 41 | 36349757 | 46.8047 | 48 | 1705718 | 45 | 1297398 |
| 42 | 36348266 | 47.4071 | 49 | 1736659 | 46 | 1301449 |
| 43 | 36346842 | 48.56 | 51 | 1770898 | 47 | 1337680 |

| | | | | | | |
|---|---|---|---|---|---|---|
| 44 | 28946844 | 53.6218 | 50 | 1771530 | 49 | 1368668 |
| 45 | 36348615 | 54.226 | 52 | 1778025 | 51 | 1403203 |
| 46 | 36348073 | 55.1908 | 54 | 1783089 | 50 | 1403836 |
| 47 | 36347957 | 55.5043 | 53 | 1783154 | 52 | 1410350 |
| 48 | 28947218 | 57.2907 | 55 | 1802152 | 55 | 1434468 |
| 49 | 36347973 | 57.9217 | 56 | 1821362 | 56 | 1453687 |
| 50 | 36345694 | 58.2313 | 57 | 1847612 | 57 | 1479940 |
| 51 | 28946592 | 58.5399 | 58 | 1868300 | 58 | 1500599 |
| 52 | 36346887 | 59.7748 | 59 | 1950748 | 59 | 1539070 |
| 53 | 36346880 | 60.3845 | 60 | 2010851 | 60 | 1598755 |
| 54 | 28949713 | 60.9763 | 61 | 2017807 | 61 | 1605695 |
| 55 | 28945639 | 61.2704 | 62 | 2038186 | 62 | 1626318 |
| 56 | 36351366 | 61.5689 | 63 | 2089372 | 63 | 1677534 |
| 57 | 36348063 | 62.1677 | 64 | 2126574 | 64 | 1714814 |
| 58 | 36346857 | 63.6344 | 67 | 2220873 | 65 | 1730150 |
| 59 | 28948072 | 64.5016 | 68 | 2267317 | 67 | 1809061 |
| 60 | 28947836 | 64.8093 | 69 | 2294491 | 68 | 1855476 |
| 61 | 36348355 | 66.4492 | 70 | 2328147 | 69 | 1882661 |
| 62 | 36346848 | 67.7193 | 71 | 2406014 | 70 | 1916192 |

| | | | | | | |
|---|---|---|---|---|---|---|
| 63 | 36348208 | 68.6096 | 73 | 2417726 | 71 | 1994076 |
| 64 | 36347608 | 68.9108 | 72 | 2450723 | 73 | 2005786 |
| 65 | 36347753 | 69.8633 | 75 | 2490539 | 72 | 2038648 |
| 66 | 36346922 | 71.1254 | 76 | 2540344 | 75 | 2077775 |
| 67 | 36349645 | 76.5357 | 77 | 2630739 | 76 | 2127596 |
| 68 | 28948182 | 77.7264 | 78 | 2662166 | 77 | 2219650 |
| 69 | 36348399 | 79.2466 | 79 | 2670938 | 78 | 2251098 |
| 70 | 36349475 | 81.7409 | 80 | 2687972 | 79 | 2259874 |
| 71 | 36345949 | 82.3451 | 82 | 2752741 | 80 | 2276908 |
| 72 | 36348715 | 82.9741 | 83 | 2786347 | 81 | 2285865 |
| 73 | 36349012 | 86.4279 | 84 | 2816173 | 82 | 2341676 |
| 74 | 100313000 | 87.0358 | 85 | 2859887 | 83 | 2375268 |
| 75 | 36345953 | 88.2518 | 86 | 2859887 | 84 | 2405374 |
| 76 | 28947915 | 89.7448 | 89 | 2880221 | 85 | 2449172 |
| 77 | 28946305 | 90.7127 | 87 | 2882883 | 86 | 2449172 |
| 78 | 36349669 | 91.9868 | 88 | 2907544 | 89 | 2469505 |
| 79 | 36347633 | 92.2827 | 92 | 3006058 | 87 | 2472167 |
| 80 | 28949110 | 92.5876 | 94 | 3034660 | 88 | 2496874 |
| 81 | 36351920 | 94.3741 | 97 | 3042427 | 91 | 2559876 |

| | | | | | | |
|---|---|---|---|---|---|---|
| 82 | 28948986 | 95.5405 | 95 | 3042492 | 92 | 2595227 |
| 83 | 36348137 | 96.7068 | 96 | 3042492 | 94 | 2624403 |
| 84 | 28949142 | 96.9942 | 99 | 3066444 | 97 | 2632173 |
| 85 | 28945690 | 97.3097 | 98 | 3090586 | 95 | 2632238 |
| 86 | 36348529 | 98.6172 | 100 | 3123264 | 96 | 2632238 |
| 87 | 36346379 | 98.9429 | 104 | 3165953 | 100 | 2712990 |
| 88 | 36346223 | 99.5902 | 102 | 3188012 | 101 | 2726629 |
| 89 | 28945362 | 101.0655 | 105 | 3416220 | 103 | 2726694 |
| 90 | 36349242 | 102.3119 | 107 | 3416220 | 104 | 2757798 |
| 91 | 36348023 | 103.2644 | 106 | 3449474 | 102 | 2780047 |
| 92 | 36347962 | 103.8705 | 109 | 3473998 | 108 | 2792967 |
| 93 | 36346501 | 104.4502 | 110 | 3488885 | 105 | 2801332 |
| 94 | 28947578 | 104.7418 | 113 | 3547083 | 107 | 2801332 |
| 95 | 36350066 | 105.0523 | 112 | 3551874 | 106 | 2834582 |
| 96 | 28946719 | 105.3781 | 111 | 3801870 | 109 | 2859109 |
| 97 | 36348103 | 107.3335 | 114 | 3818573 | 110 | 2873965 |
| 98 | 28945799 | 109.8356 | 115 | 3853964 | 113 | 2932063 |
| 99 | 28946877 | 112.6422 | 116 | 3877130 | 112 | 2936833 |
| 100 | 36346188 | 113.9715 | 117 | 3902907 | 111 | 2944457 |

| | | | | | | |
|---|---|---|---|---|---|---|
| 101 | 36346128 | 114.3082 | 118 | 3917272 | 114 | 2961119 |
| 102 | 36348233 | 114.6393 | 119 | 3945395 | 115 | 2988569 |
| 103 | 36346078 | 114.9405 | 120 | 3984365 | 117 | 3028236 |
| 104 | 28948653 | 118.0598 | 125 | 4053742 | 118 | 3042604 |
| 105 | 28948236 | 118.3704 | 127 | 4073344 | 119 | 3070733 |
| 106 | 28948866 | 118.6553 | 121 | 4087083 | 120 | 3109705 |
| 107 | 36350815 | 118.9556 | 129 | 4161369 | 125 | 3169251 |
| 108 | 28945408 | 120.1791 | 122 | 4217916 | 127 | 3185912 |
| 109 | 36346366 | 121.4025 | 123 | 4253394 | 121 | 3199655 |
| 110 | 28947845 | 121.9724 | 124 | 4253394 | 129 | 3255916 |
| 111 | 36349403 | 122.5897 | 130 | 4299074 | 122 | 3312550 |
| 112 | 36350772 | 123.2089 | 131 | 4307091 | 123 | 3359795 |
| 113 | 36347530 | 123.4962 | 133 | 4350354 | 124 | 3359795 |
| 114 | 28945953 | 123.802 | 134 | 4366301 | 130 | 3405421 |
| 115 | 36348446 | 124.4452 | 135 | 4376246 | 131 | 3413437 |
| 116 | 36350281 | 125.0494 | 136 | 4407795 | 133 | 3456692 |
| 117 | 28947561 | 125.9424 | 137 | 4436378 | 134 | 3472627 |
| 118 | 36350592 | 127.1366 | 138 | 4439539 | 135 | 3482608 |
| 119 | 28947989 | 129.8666 | 139 | 4478154 | 136 | 3514299 |

| | | | | | | |
|---|---|---|---|---|---|---|
| 120 | 28945577 | 130.8408 | 140 | 4517649 | 137 | 3542855 |
| 121 | 36347002 | 131.8118 | 141 | 4532179 | 138 | 3546018 |
| 122 | 36349059 | 132.4528 | 142 | 4533039 | 139 | 3582547 |
| 123 | 36350626 | 133.727 | 143 | 4543429 | 140 | 3622500 |
| 124 | 28946888 | 134.0465 | 144 | 4567073 | 141 | 3637071 |
| 125 | 36349467 | 134.3691 | 145 | 4610156 | 142 | 3637931 |
| 126 | 36345948 | 134.6845 | 147 | 4620139 | 143 | 3648316 |
| 127 | 36350012 | 135.3175 | 146 | 4633564 | 144 | 3671931 |
| 128 | 36346496 | 135.6142 | 148 | 4672269 | 145 | 3715021 |
| 129 | 28946281 | 136.7738 | 149 | 4685683 | 147 | 3724998 |
| 130 | 28946242 | 137.0645 | 150 | 4690365 | 146 | 3738435 |
| 131 | 28948060 | 138.2208 | 151 | 4708549 | 148 | 3777138 |
| 132 | 36346474 | 139.3805 | 152 | 4718953 | 149 | 3790560 |
| 133 | 36349845 | 140.5334 | 153 | 4729059 | 150 | 3795241 |
| 134 | 28948261 | 141.7458 | 154 | 4729835 | 151 | 3813428 |
| 135 | 36348402 | 143.5871 | 155 | 4783278 | 152 | 3823822 |
| 136 | 36350245 | 144.1753 | 156 | 4797615 | 153 | 3833919 |
| 137 | 28947330 | 144.4695 | 157 | 4832017 | 154 | 3834695 |
| 138 | 36348098 | 145.056 | 161 | 4884964 | 155 | 3858904 |

| | | | | | | |
|---|---|---|---|---|---|---|
| 139 | 28946586 | 146.1958 | 160 | 4886503 | 156 | 3873242 |
| 140 | 28947120 | 146.4823 | 162 | 4942204 | 157 | 3907650 |
| 141 | 28947781 | 146.7705 | 163 | 4961644 | 161 | 3960674 |
| 142 | 28947882 | 147.6377 | 165 | 5001210 | 160 | 3962213 |
| 143 | 28948352 | 148.2277 | 166 | 5030026 | 162 | 4019407 |
| 144 | 36347555 | 152.5195 | 167 | 5040777 | 163 | 4038466 |
| 145 | 36348219 | 154.3167 | 168 | 5097911 | 165 | 4078092 |
| 146 | 28946928 | 154.9265 | 169 | 5107258 | 166 | 4106882 |
| 147 | 28947197 | 155.867 | 170 | 5161999 | 167 | 4117640 |
| 148 | 28948055 | 156.7988 | 171 | 5179014 | 168 | 4174958 |
| 149 | 36350067 | 157.6585 | 172 | 5198201 | 169 | 4184317 |
| 150 | 36348499 | 158.5281 | 176 | 5206490 | 170 | 4209412 |
| 151 | 28946506 | 160.6003 | 183 | 5537699 | 171 | 4226415 |
| 152 | 36345896 | 163.6039 | 182 | 5539275 | 172 | 4245443 |
| 153 | 28949425 | 165.9082 | 173 | 5569770 | 176 | 4253735 |
| 154 | 36350687 | 166.2058 | 174 | 5633733 | 183 | 4398631 |
| 155 | 28946917 | 166.5034 | 175 | 5698404 | 182 | 4400201 |
| 156 | 28947040 | 169.5685 | 177 | 6016595 | 178 | 4457355 |
| 157 | 36346096 | 170.2985 | 178 | 6045428 | 179 | 4476097 |

| | | | | | | |
|---|---|---|---|---|---|---|
| 158 | 36349232 | 170.617 | 179 | 6064019 | 181 | 4493767 |
| 159 | 28949067 | 170.9173 | 181 | 6081848 | #N/A | |
| 160 | 28947296 | 171.2259 | 180 | 6087410 | #N/A | |
| 161 | 36346793 | 174.7574 | #N/A | 6203058 | #N/A | |
| 162 | 36348359 | 175.6584 | #N/A | | #N/A | |
| 163 | 36349494 | 175.956 | #N/A | | #N/A | |
| 164 | 28949083 | 176.2735 | #N/A | | #N/A | |
| 165 | 36350923 | 177.5396 | #N/A | | #N/A | |
| 166 | 36346807 | 178.8344 | #N/A | | #N/A | |
| 167 | 36348904 | 182.4442 | #N/A | | #N/A | |
| 168 | 28949572 | 183.0274 | #N/A | | #N/A | |
| 169 | 28947030 | 185.2094 | #N/A | | #N/A | |
| 170 | 36347736 | 185.8874 | #N/A | | #N/A | |
| 171 | 36349801 | 187.2391 | #N/A | | #N/A | |
| 172 | 36346475 | 188.1709 | #N/A | | #N/A | |
| 173 | 36350166 | 189.0719 | #N/A | | #N/A | |
| 174 | 36346514 | 189.3768 | #N/A | | #N/A | |
| 175 | 28946201 | 190.2751 | #N/A | | #N/A | |
| 176 | 100241067 | 197.3338 | #N/A | | #N/A | |

| | | | | | | |
|---|---|---|---|---|---|---|
| 177 | 28946922 | 197.9492 | #N/A | | #N/A | |
| 178 | 36348282 | 199.119 | #N/A | | #N/A | |
| 179 | 36351201 | 199.7055 | #N/A | | #N/A | |
| 180 | 28946886 | 200.9838 | #N/A | | #N/A | |
| 181 | 36346275 | 210.5131 | #N/A | | #N/A | |
| 182 | 28946413 | 210.842 | #N/A | | #N/A | |
| 183 | 28945787 | 210.843 | #N/A | | #N/A | |

#N/A Not available

Supplementary Table S3. Candidate effector genes and predicted proteins for QTL identified in this study

| QTL[a] | Reference genome | Peak marker position[b] (bp) | Start[c] | End[d] | Gene ID[e] | Effector/non[f] effector (EffectorP) | Expression[g] profile (Ismail and Able 2016, 2017) | Protein family (pfam) | Description (pfam) | Clan (pfam) | Protein (pfam)[h] |
|---|---|---|---|---|---|---|---|---|---|---|---|
| *USQV2* | *W1-1* | 337069 | 317069 | 357069 | NA | NA | | | | | NA |
| *USQV5* | | 3719823 | 3699823 | 3739823 | PTTW11_06575 | Effector | NA | NA | NA | NA | Hypothetical protein |
| | | | | | PTTW11_06576 | Non effector | | | | | Hypothetical protein |
| | | | | | PTTW11_06577 | Effector | Effector | Thioredoxin | Thioredoxin | CL0172 | Thioredoxin |
| | | | | | PTTW11_06578 | Non effector | | | | | Malate dehydrogenase |
| | | | | | PTTW11_06579 | Non effector | | | | | Zeta-crystallin |
| | | | | | PTTW11_06580 | Non effector | | | | | Hypothetical protein |
| | | | | | PTTW11_06581 | Non effector | | | | | Hypothetical protein |
| | | | | | PTTW11_06582 | Non effector | | | | | Nucleotid-trans domain containing protein |
| | | | | | PTTW11_06583 | Non effector | | | | | APG6 domain containing protein |
| | | | | | PTTW11_06584 | Non effector | | | | | Aldehyde dehydrogenase |
| | | | | | PTTW11_06585 | Non effector | Effector | Peptidase A4 family | Peptidase A4 family | CL0004 | Acid protease |
| | | | | | PTTW11_06586 | Non effector | | | | | Integral membrane protein |
| | | | | | PTTW11_06587 | Non effector | | | | | BCAS2 family protein |
| | | | | | PTTW11_06588 | Non effector | | | | | Neutral ceramidase |
| | | | | | PTTW11_06589 | Non effector | | | | | Dipeptidase domain containing protein |
| *USQV8* | | 6007171 | 5987171 | 6027171 | NA | | | | | | NA |
| *USQNB5.1* | *SG1* | 313831 | 293831 | 333831 | PTMSG1_05073 | Effector | NA | | CFEM domain | | Hypothetical protein |
| | | | | | PTMSG1_05074 | Non effector | | | | | Hypothetical protein |
| | | | | | PTMSG1_05075 | Non effector | | | | | Hypothetical protein |
| | | | | | PTMSG1_05076 | Non effector | | | | | Hypothetical protein |
| | | | | | PTMSG1_05077 | Non effector | | | | | Hypothetical protein |
| | | | | | PTMSG1_05078 | Non effector | | | | | Hypothetical protein |
| | | | | | PTMSG1_05079 | Non effector | | | | | Hypothetical protein |
| | | | | | PTMSG1_05080 | Non effector | | | | | Hypothetical protein |

| | | | | | | | | | | | |
|---|---|---|---|---|---|---|---|---|---|---|---|
| | | | | | PTMSG1_05081 | Non effector | | | | | Hypothetical protein |
| | | | | | PTMSG1_05082 | Non effector | | | | | Hypothetical protein |
| | | | | | PTMSG1_05083 | Non effector | | | | | Hypothetical protein |
| | | | | | PTMSG1_05084 | Non effector | | | | | Hypothetical protein |
| *USQNB5.2* | | 4362659 | 4342659 | 4382659 | NA | NA | | | | | NA |
| | | 4413961 | 4393961 | 4433961 | | | | | | | |
| *USQV9* | | 876223 | 856223 | 896223 | PTMSG1_08700 | Non effector | | | | | Hypothetical protein |
| | | | | | PTMSG1_08701 | Non effector | | | | | Methyltransferase UbiE |
| | | | | | PTMSG1_08702 | Non effector | | | | | Hypothetical protein |
| | | | | | PTMSG1_08703 | Non effector | | | | | mfs multidrug transporter |
| | | | | | PTMSG1_08704 | Non effector | | | | | Structural maintenance of chromosomes protein |
| | | | | | PTMSG1_08705 | Non effector | | | | | DUF2838 domain containing protein |
| | | | | | PTMSG1_08706 | Non effector | | | | | NAD-binding-8 multi-domain protein |
| | | | | | PTMSG1_08707 | Non effector | | | | | Separin |
| | | | | | PTMSG1_08708 | Non effector | | | | | Git3 multi-domain protein |
| *USQNB11* | | 318802 | 250568 | 394230 | PTMSG1_09701 | Non effector | | | | | Zuotin |
| | | | | | PTMSG1_09702 | Non effector | | | | | Leucine zipper protein |
| | | | | | PTMSG1_09703 | Non effector | | | | | Serine/threonine-protein kinase SRPK2 |
| | | | | | PTMSG1_09704 | Non effector | | | | | Acetolactate synthase |
| | | | | | PTMSG1_09705 | Non effector | | | | | TPR-16 domain containing protein |
| | | | | | PTMSG1_09706 | Non effector | | | | | Hypothetical protein |
| | | | | | PTMSG1_09707 | Non effector | | | | | SWAP multi-domain protein |
| | | | | | PTMSG1_09708 | Non effector | | | | | Hypothetical protein |
| | | | | | PTMSG1_09709 | Non effector | | | | | Hypothetical protein |
| | | | | | PTMSG1_09710 | Effector | | | | | Dolichol-phosphate mannosyltransferase |
| | | | | | PTMSG1_09711 | Effector | | | | | Hypothetical protein |
| | | | | | PTMSG1_09712 | Non effector | | | | | UbiH 2-polyprenyl-6-methoxyphenol hydroxylase |
| | | | | | PTMSG1_09713 | Non effector | | | | | Hypothetical protein |
| | | | | | PTMSG1_09714 | Effector | | | | | Hypothetical protein |
| | | | | | PTMSG1_09715 | Non effector | | | | | Hypothetical protein |

| | | | | | | | | | | | |
|---|---|---|---|---|---|---|---|---|---|---|---|
| | | | | | PTMSG1_09716 | Effector | | | | | Hypothetical protein |
| *USQV12* | | 128854 | 108854 | 148854 | PTMSG1_10196 | Non effector | | | | | Hypothetical protein |
| | | | | | PTMSG1_10197 | Effector | | | | | Hypothetical protein |
| | | | | | PTMSG1_10198 | Effector | | | | | Hypothetical protein |
| | | | | | PTMSG1_10199 | Non effector | | | | | Hypothetical protein |
| | | | | | PTMSG1_10200 | Non effector | | | | | zf-MIZ multi-domain protein |
| | | | | | PTMSG1_10201 | Non effector | | | | | Polarized growth protein Boi2 |
| | | | | | PTMSG1_10202 | Effector | | | | | Hypothetical protein |
| | | | | | PTMSG1_10203 | Non effector | | | | | Glycosyltransferase family 31 protein |
| | | | | | PTMSG1_10204 | Effector | | | | CL0199 | Glycoside hydrolase family 45 protein |
| | | | | | PTMSG1_10205 | Non effector | | | | | Sulfate permease |
| | | | | | PTMSG1_10206 | Non effector | | | | | Hypothetical protein |
| | | | | | PTMSG1_10207 | Non effector | | | | | Hypothetical protein |
| | | | | | PTMSG1_10208 | Non effector | | | | | Hypothetical protein |
| | | | | | PTMSG1_10209 | Non effector | | | | | Hypothetical protein |
| | | | | | PTMSG1_10210 | Non effector | | | | | Maf Nucleotide-binding protein implicated in inhibition septummation |
| | | | | | PTMSG1_10211 | Non effector | | | | | NADP-dependent leukotriene B4 12-hydroxydehydrogenase |
| *USQNB12* | | 1916192 | 1896192 | 1936192 | NA | NA | | | | | NA |

[a] QTL-Quantitative trait loci identified from this study

[b] Peak marker position-Peak marker position of the QTL corresponding reference genome

[c] Start- Stating position of the QTL

[d] End - Ending position of the QTL

[e] Gene ID- Candidate gene identity base on NCBI repository

[f] Effector/non effector- identification of the candidate gene as an effector by EffectorP

[g] Expression profile -identification of the candidate gene as an effector by Ismail and Able 2016, 2017

[h] pfam- The protein family database
